# Genome-Wide Development and Characterization of 169 gSSR Markers in the Invasive Plant *Xanthium strumarium* L.

**DOI:** 10.3390/plants14223522

**Published:** 2025-11-18

**Authors:** Junshuang Yin, Qingyao Bai, Yiting Mao, Hui Min, Chunsha Zhang, Yibo Sun, Xiaojia Zhang, Yulong Feng

**Affiliations:** College of Bioscience and Biotechnology, Shenyang Agricultural University, Shenyang 110065, China

**Keywords:** polymorphism, SSR markers, taxonomy, *Xanthium*

## Abstract

*Xanthium strumarium* L. is a plant species native to North America; however, it has become a serious invasive threat in northern China due to its great environmental adaptability in the colonized regions. Therefore, elucidating its genetic traits is crucial to understanding its adaptive success. Simple sequence repeats (SSRs) comprise 1–6 nucleotides within plant genomes, which are available for evaluating the level of plant genetic diversity. However, the comprehensive analysis of high-coverage SSR markers in *Xanthium* is limited. This study identified 450,847 SSR loci in the *X. strumarium* genome. The number of SSR loci decreased with increasing SSR length within the range of 10–100 bp. Dinucleotide repeats constituted the majority (49.81%), totaling 221,154, with AT/TA motifs being the most frequent (66.62%). We developed 169 gSSR markers covering all *X. strumarium* chromosomes, with 5–15 markers per chromosome. Moreover, the number of different alleles (Na), number of effective alleles (Ne), Shannon’s information index (I), observed heterozygosity (Ho), expected heterozygosity (He), and polymorphism information content (PIC) were varied from 1.2 to 3.3, 1.077 to 2.385, 0.087 to 0.903, 0 to 1, 0.056 to 0.558, and 0.161 to 0.853, respectively. This marks the first systematic development of high-coverage SSR markers in the genus *Xanthium*, which increases the number of available SSR markers and reveals the molecular foundation of adaptation to invasion.

## 1. Introduction

*Xanthium strumarium* L. is native to North America but has become one of the most successful invasive species in many parts of the world, including northern China [1]. *X. strumarium* has key physiological differences to its native Chinese counterpart, *Xanthium sibiricum* L., which enable it to acquire nitrates at a substantially higher rate, allowing for higher growth plasticity [2,3,4,5]. Additionally, this enhanced ability of *X. strumarium* influences local legume plants to rely more heavily on nitrate nitrogen source [6]. This superior ability of *X. strumarium* to uptake and use nitrate allows it to outcompete native species for resources and available growth space. These findings provide a molecular explanation for the resource utilization hypothesis [3,5]. Research into its intricate underlying mechanism, beyond nitrogen resource utilization, remains largely unexplored. Furthermore, the colonization of northern China by the invasive species of *X. strumarium* has complicated traditional taxonomic classifications, making the agreed number of *Xanthium* species in China controversial [7,8]. Beyond traditional classification methods, utilizing molecular approaches to analyze the complex genetic background of *Xanthium* species offers a promising alternative.

Simple sequence repeats (SSRs), which consist of 1–6 nucleotides and exhibit high polymorphism, are ubiquitous in plant genomes [9,10]. SSRs have been widely used in various fields, including phylogenetic analysis, genetic diversity studies, marker-assisted selection, gene identification, and germplasm characterization [1,11,12,13,14,15,16]. In recent decades, SSR technology has been increasingly utilized in both staple and cash crops, serving as a pivotal tool for genetic improvement and species conservation [13,14,15,16].

SSRs in plants are currently categorized into genomic SSRs (gSSRs) and expression sequence tag SSRs (EST-SSRs) based on the sequences from which they are derived [14,17]. gSSRs, developed from genomic DNA, cover both coding and non-coding regions. They are highly polymorphic and widely distributed across the genome. Comparatively, EST-SSRs are derived exclusively from regions of expressed genes that have clear functional annotations. Each type of SSR has its strengths and limitations: gSSRs offer comprehensive genomic coverage and strong versatility, particularly suitable for wild or non-model species, but at a higher cost [14,18,19]. EST-SSRs can be linked to functional genes but rely on transcriptome data for development. They are less polymorphic but are comparatively more cost-effective [16,20].

Previous studies have developed eight pairs of inter-simple sequence repeat (ISSR) markers to assess genetic diversity and differentiation analyses in *X. italicum* Moretti [1]. Raman et al. [8] assembled the chloroplast genome of *X. spinosum* L. and identified 701 chloroplast SSRs. The quantity of these SSRs was relatively low, indicating that they were in an early stage of development and validation. In this study, we systematically developed and validated 169 gSSR markers using data from our unpublished sources on *X. strumarium*. These gSSRs provide full chromosomal coverage, overcoming limitations of organelle-specific or low-density markers. Moreover, developing an SSR marker system for the invasive species of *X. strumarium* holds dual significance. Firstly, identifying and understanding the genetic background and molecular bases of *X. strumarium*’s success help researchers uncover general mechanisms of invasion adaptation in plants. This is crucial for developing management and prevention strategies for invasive species. Secondly, compared to its native congener *X. sibiricum*, *X. strumarium* exhibits specific invasive traits, notably an enhanced capacity for nitrate uptake [2,3,4,5]. An SSR marker system can help identify the genes that contribute to these physiological differences. Our objectives in this study included (1) systematically identifying genome-wide SSR loci in *X. strumarium*; (2) developing and validating a set of SSR markers covering all chromosomes; and (3) evaluating their utility in genetic diversity analysis and molecular classification within *X. strumarium*, with particular emphasis on applications in invasion genetics.

## 2. Results

### 2.1. Analysis of SSR Repetition Frequency and Length

A total of 450,847 SSRs were isolated in the genome of *X. strumarium*. These SSRs had an average distance of 6.91 kb and an occurrence frequency of 0.24%. Chromosome 0 harbored the highest number of SSRs (33,645, accounting for 7.46% of the total), whereas Chromosome 9 had the lowest number (18,346, accounting for 4.07%) (Table S1 and Figure S1).

**Figure 1:**
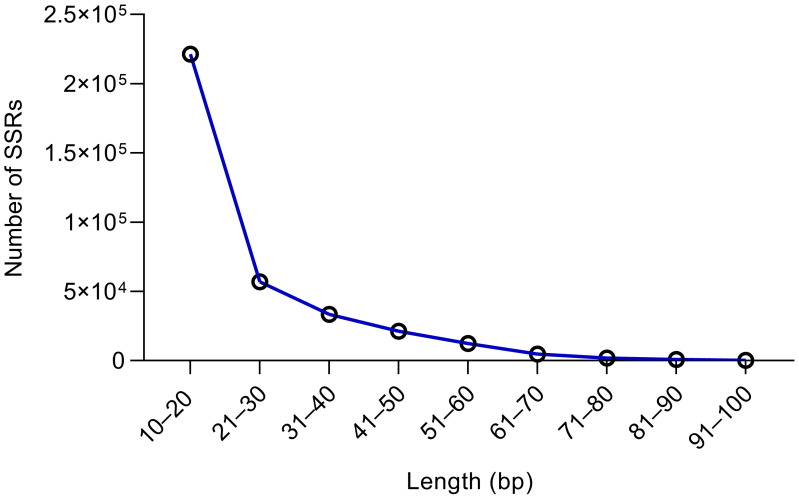
SSR length (10–100 bp) distribution information in *Xanthium strumarium*.

The average length of the SSR motifs was 21.32 bp, with lengths ranging from 12 to 2994 bp. Almost half of the SSRs (49.11%), a total of 221,425, were found to have a length of within 20 bp (Figure 1). The least abundant SSRs (0.06%) were those between 91 and 100 bp in length, totaling only 288. SSRs longer than 100 bp represented 21.53% of the overall total, yet their distribution varied significantly across different lengths. Generally, the number of SSR loci decreased with increasing SSR length within the 10–100 bp range (Figure 1 and Table 1).

### 2.2. Characteristics of SSRs Predicted in the Genome of X. strumarium

We further analyzed the predicted SSR characteristics in the *X. strumarium* genome. Among the various types, dinucleotide repeats exhibited the smallest average distribution distance of 10.17 kb, while hexanucleotide repeats had the largest distance of 442.90 kb. Dinucleotide repeats represented the highest proportion, accounting for 49.81% of all repeat types, with trinucleotide repeats following at 42.43%. The remaining repeat types together accounted for only 7.76% (Table 2). Therefore, dinucleotide and trinucleotide repeats were the most abundant genome of *X. strumarium*.

**Figure 2:**
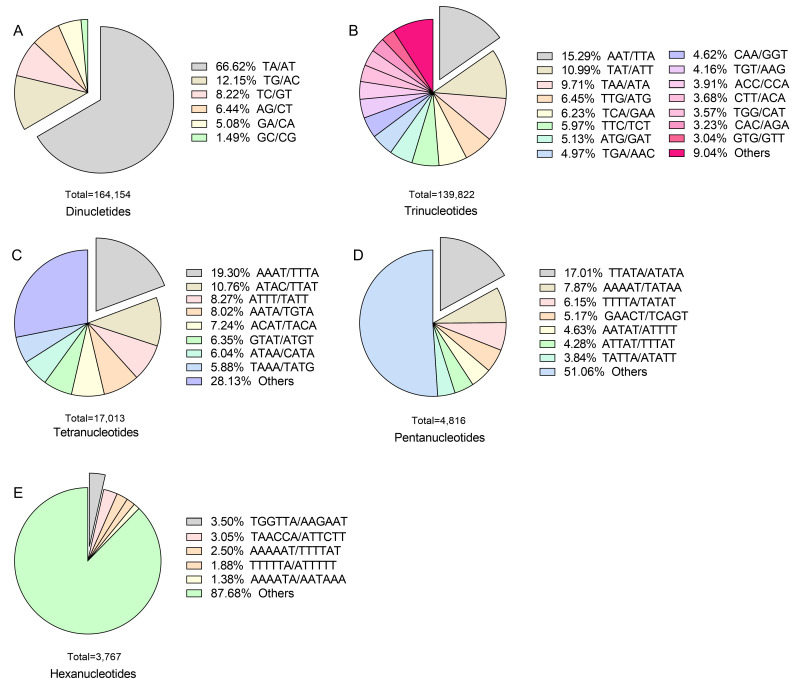
Distribution of motif types in the whole genomes of *X. strumarium*. SSR motif types include the following five categories: Dinucleotide (**A**), Trinucleotide (**B**), Tetranucleotide (**C**), Pentanucleotide (**D**), and Hexanucleotide (**E**). The differently colored boxes in the pie graph represent the relative sizes of the proportions of different motifs.

Regarding SSR motif types, within dinucleotide repeats, the AT/TA motif was the most frequent (66.62%), followed by AC/TG (12.15%) and GT/TC (8.22%), showing a relatively uneven distribution (Figure 2A). For trinucleotide repeats, AAT/TTA was the most prevalent motif (15.29%), followed by TAT/ATT (10.99%) and TAA/ATA (9.71%) (Figure 2B). Among tetranucleotide repeats, AAAT/TTTA was the most frequent (19.30%), followed by ATAC/TTAT (10.76%) and ATTT/TATT (8.27%) (Figure 2C). In pentanucleotide and hexanucleotide repeats, TTATA/ATATA (17.01%) and TGGTTA/AAGAAT (3.5%) were the most abundant, respectively (Figure 2D,E). Overall, the AT/TA motif within dinucleotide repeats was the most prevalent in *X. strumarium*, and this motif became the subject of further analysis and validation.

### 2.3. Identification and Characterization of 169 gSSR Markers with Polymorphism Across 18 Chromosomes

**Figure 3:**
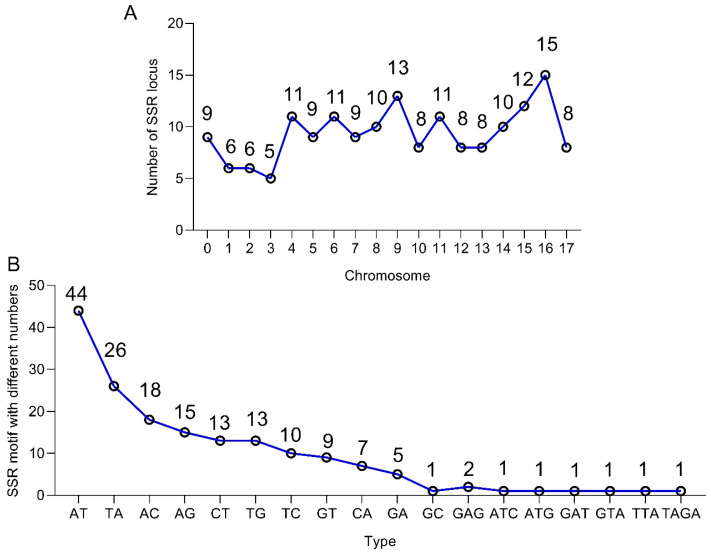
Distribution information of 169 SSRs locus in *X. strumarium*. (**A**) The number of SSR loci with different chromosomes. (**B**) The different number of SSR motifs.

To further substantiate the predicted SSRs in the *X. strumarium* genome, we selected 100 SSR loci from each chromosome for verification (a total of 1800 loci). A total of 169 loci were isolated across 18 chromosomes, for which corresponding SSR markers were developed (Figure S2 and Table S2). The majority of chromosomes (excluding chromosomes 1, 2, and 3) harbored more than 8 SSR loci, with a range of 5 to 15 per chromosome (Figure 3A). The most abundant motifs found in the validated SSR sequences were AT and TA. The number of AT and TA motifs counted was 44 and 26, respectively, while other motifs were found in numbers less than 19 (Figure 3B and Table S3). Dinucleotide repeats were the most common among all repeat types, amounting to 161, followed by trinucleotide and tetranucleotide repeats, which had only 7 and 1, respectively (Figure 3B and Table S3), which was consistent with our previous predictions (Table 2 and Figure 2). These screened SSR lengths ranged from 12 bp to 54 bp, SSR motif repetition numbers were between 4 and 27, and overall SSR loci lengths were between 80 bp and 214 bp (Table S2).

### 2.4. gSSR Marker Assay and Their Informativeness

To further validate the polymorphisms associated with 169 gSSR markers, we randomly selected one pair of SSR primers from each chromosome to examine 160 *X. strumarium* species and 40 *X. sibiricum* species. All 18 primer pairs were successfully amplified. Table 3 lists the genetic diversity indices calculated from the 18 SSR markers for the 200 species. The number of different alleles (Na) ranged from 1.2 to 3.3, with an average value of 2.006. The number of effective alleles (Ne) ranged from 1.077 to 2.385, averaging 1.730. Shannon’s information index (I) ranged from 0.087 to 0.903, with an average value of 0.567. Observed heterozygosity (Ho) ranged from 0 to 1, averaging 0.672. Expected heterozygosity (He) ranged from 0.056 to 0.558, averaging 0.377. Polymorphic information content (PIC) ranged from 0.161 to 0.853, averaging 0.620. These results demonstrate that the 18 SSR markers can be used to effectively evaluate polymorphisms within the *X. strumarium* and *X. sibiricum* species.

## 3. Discussion

Previous studies have shown that SSR repeat types primarily include mononucleotide, dinucleotide, and trinucleotide repeats, and their relative abundance varies significantly among different species and even within different genomic regions of the same species [11,12,17,19,21,22]. An analysis of *X. strumarium* identified a total of 164,154 dinucleotide repeats, accounting for 49.81% of the total (Table 2). From our findings, dinucleotide repeats were the most predominant type in *X. strumarium*, which is consistent with the reports on modern sugarcane cultivars, *Brassica* spp., and *Weigela* cultivars [11,12,19]. This has also been observed in other Asteraceae species such as *Chrysanthemum sensu lato* [10,14], but differs from findings from *Chimonanthus praecox* L., *Elymus sibiricus* L., and *Elymus breviaristatus* (Keng) Keng f. [17,21,22]. Among these dinucleotide repeats, TA/AT motif repeats were the most common, accounting for 66.62% (Figure 2A), which aligns with observations made in *Brassica* spp. and *Phaseolus vulgaris* L. [19,23]. Additionally, subsequent validation of SSR motifs further confirmed this preponderance (Tables S2 and S3). AT base pairs are linked by two hydrogen bonds, while GC base pairs are bonded by three [24]. This suggests that TA/AT motif repeats might be energetically less demanding for organisms, although some species were also identified to have AG/GA and AG/CT as the primary dinucleotide repeats [11,12]. In *X. strumarium*, the prevalence of TA/AT motifs potentially signifies an evolutionary balance between energy efficiency and mutational stability. The reduced energy requirement for DNA replication in AT-rich regions, due to a lower number of hydrogen bonds [24,25], could confer an advantage for *X. strumarium* in rapidly colonizing environments. Additionally, trinucleotide repeats constitute approximately 46% in *X. strumarium*, with AAT/TTA identified as the most prevalent motif sequence (Figure 2B), which is different from AAG/CTT in *Chrysanthemum × morifolium* Ramat. and CCG/CGG in *C. praecox*. These differences may arise from species-specific evolutionary selection processes affecting SSR sequences [26].

SSRs within plant genomes are widely used in plant genetic analysis due to their high abundance, genetic diversity, and high variability. They are valuable for identifying genetic differences and are applied in gene mapping, identifying genetic diversity, and germplasm identification [8,15,16,17,27,28,29]. Previous studies in species such as *C.* × *morifolium* and *C. praecox* reported EST-SSR loci ranging from thousands to tens of thousands, with successfully developed SSR markers in the single to double digits [14,16,17]. In contrast, gSSRs provide broader coverage (both coding and non-coding regions). Non-conserved regions exhibit less conservation and greater variation across species within the same genus compared to conserved regions. This high variability makes gSSR markers more polymorphic and better for distinguishing closely related individuals or populations within a species [11,12]. In this study, 450,847 SSRs were identified in *X. strumarium*, covering its entire genome (Table S1 and Figure S1). Furthermore, we successfully developed 169 gSSR markers for the first time, spanning all 18 chromosomes (Figure S2 and Figure 3A, and Table S2). The polymorphic rate in both *X. strumarium* and *X. sibiricum* was approximately 9.4% (169/1800), comparable to the 9.3% found in *C. praecox* [17] but lower than 32.62% in 12 sugarcane materials [30].

We observed that the PIC values in our study reached up to 0.853 (Table 3), showing high genetic diversity in *X. strumarium*. This value is comparatively higher than in Asteraceae: invasive *Ambrosia artemisiifolia* L. (0.21–0.82) [31]; non-invasive *C.* × *morifolium* (0.38–0.50) [32]; and *Gerbera hybrida* Hort. (0.13–0.80) [33]. The substantial variation in SSR polymorphic rates and PIC values among species may have resulted from multiple factors, including species evolutionary history (phylogenetic relationship, reproductive strategies), genomic characteristics (repeat unit stability, ploidy level), and SSR screening methods (primer specificity, sample selection) [17,30,34,35]. The invasive biology of *X. strumarium* can be linked to its high PIC value. During long-distance colonization, invasive plants frequently retain high genetic diversity to adapt to heterogeneous environments. Furthermore, we speculate that during its expansion, *X. strumarium* probably encountered strong selective pressures and heterogeneous environments frequently, as shown by its high PIC value. High PIC values indicate that a genetically diverse population offers broader raw materials for natural selection, which enabled *X. strumarium* to rapidly adapt to abiotic stresses (e.g., low nitrogen) and biotic interactions (e.g., competition), ultimately facilitating successful invasion.

Conclusively, the development of 169 gSSR markers in this study signifies a significant advancement over earlier research on the genus *Xanthium*. Firstly, it achieves full chromosomal coverage in *X. strumarium* for the first time, overcoming the limitation of previous chloroplast genome markers in *X. spinosum*, which could only reflect uniparental genetic information [8]. Secondly, compared to the limited SSR markers used in earlier studies on population genetic differentiation in *Xanthium* [1], this marker system has the potential to significantly enhance the resolution of fine genetic variations among closely related species (for example, *X. sibiricum* and *X. strumarium*, or other *Xanthium* species) by utilizing high-density loci. Thirdly, it provides key technical support for the analysis of population genetic structure, inference of invasion routes, and the genetic mapping of invasion-related traits (for example, rapid growth and resource competition) of the invasive species of *X. strumarium*. These markers can facilitate future comparisons of genetic differences between invasive and native populations and help identify gene loci linked to invasion adaptability.

## 4. Materials and Methods

### 4.1. Plant Materials and gDNA Extraction

*X. strumarium* was collected from Kangping, Liaoning, China (123°17′41.92″ E, 42°42′57.25″ N). SSR markers were developed using both *X. strumarium* and the native congeneric species *X. sibiricum*, which were conserved by the biological invasion team at Shenyang Agricultural University. Following a 4 °C vernalization for 2–3 days, seeds of *X. strumarium* and *X. sibiricum* were then germinated in a growth chamber for 2–3 days at 28 °C with a light intensity of 200 μmol/m^2^·s, maintained under a 12 h light/12 h dark cycle. Total genomic DNA was extracted from germinated seedlings of both species using the CTAB method [36]. The extracted DNA was visualized via agarose gel electrophoresis to confirm normal banding patterns, and DNA concentrations were then measured using a NanoDrop 1000 spectrophotometer (Thermo Fisher Scientific, Waltham, MA, USA). The DNA concentrations of *X. strumarium* and *X. sibiricum* were both adjusted to 80 ng/µL.

### 4.2. SSR Screening

**Figure 4:**
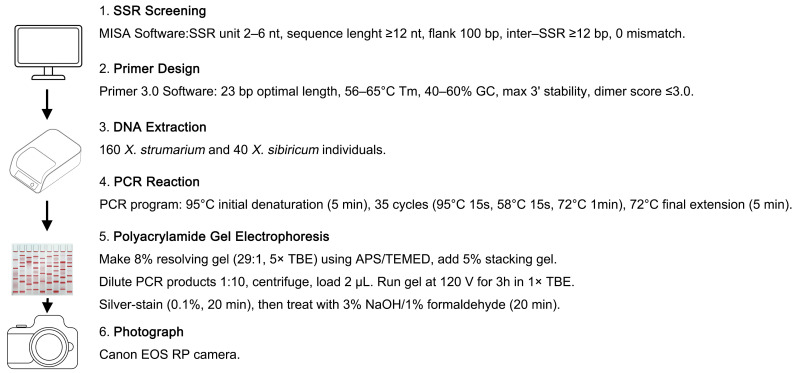
A schematic workflow of the gSSR marker development process for *X. strumarium* and *X. sibiricum*.

We conducted an exhaustive genome-wide exploration for SSR within our unpublished genome of *X. strumarium* utilizing the Microsatellite Identification Tool (MISA) software (web-based version) [37]. The initial screening criteria were carefully formulated as follows: a minimal SSR repeat unit length of 2 nucleotides, a maximal length constraint of 6 nucleotides, a minimum SSR sequence length threshold of 12 nucleotides, a flanking sequence length of 100 base pairs, and a minimum inter-SSR distance of 12 base pairs [38,39,40]. The mismatch threshold was set to 0, and flanking sequences with low-complexity regions or N bases were excluded (Figure 4). All remaining parameters were standardized to their operational software-default settings to ensure consistency and rigor in our methodological approach.

### 4.3. Primer Design and PCR Reaction

Primers were designed for the flanking sequences of SSR loci identified in the *X. strumarium* genome using Primer 3.0 software [41]. Primer design followed specific parameters set as: an optimal length of 23 bp, an annealing temperature range of 56–65 °C, a GC content between 40% and 60%, a maximum 3′ stability with *Δ*G ≤ −9 kcal/mol, and a dimer score of ≤3.0 (Figure 4). During the process of selecting flanking regions for primer design, we ensured that the sequences were unique within the genome to avoid cross-reactivity with other genomic regions. Additionally, secondary structures that could disrupt primer binding and PCR efficiency, such as hairpins or self-dimers, were avoided. The remaining parameters were configured to the default values of the software. Selected SSR primers from each chromosome were used for PCR analysis with four samples from two *X. strumarium* and two *X. sibiricum* species. PCR reactions were performed according to the manufacturer’s instructions for EasyTaq^®^ DNA Polymerase (AP111-01) (TransGen Biotech, Beijing, China). The PCR program consisted of an initial denaturation at 95 °C for 5 min, followed by 35 cycles of PCR (95 °C for 15 s, 58 °C for 15 s, 72 °C for 1 min), and a final extension at 72 °C for 5 min (Figure 4).

### 4.4. Polyacrylamide Gel Electrophoresis (PAGE) Protocol

An 8% resolving gel (acrylamide:bis-acrylamide = 29:1) containing 5× TBE buffer was prepared and polymerized with the addition of 10% APS and TEMED, followed by overlaying with a 5% stacking gel. PCR products were mixed with formamide loading buffer (10×) at a ratio of 1:10 and centrifuged at 4000 rpm. Subsequently, 2 μL of the mixture of PCR products was loaded onto the gel and electrophoresed at a constant voltage of 120 V for 3 h in 1× TBE buffer. Silver staining was performed for 20 min using a 0.1% solution, then it was immersed in a 3% NaOH solution containing 1% formaldehyde for another 20 min. The reaction was terminated by adding a small amount of boric acid [11,42,43]. Finally, the gel was placed in ddH_2_O and photographed using a Canon EOS RP camera (Canon Inc., Tokyo, Japan) for documentation (Figure 4).

### 4.5. Analysis of gSSR Markers

The genetic diversity of 160 *X. strumarium* species and 40 *X. sibiricum* species was reflected by newly developed gSSR markers. These species were amplified by the selective gSSR primers. The genetic diversity indices, including the number of different alleles (Na), the number of effective alleles (Ne), Shannon’s information index (I), observed heterozygosity (Ho), and expected heterozygosity (He), were computed using GenAlEx 6.5 software [44]. Polymorphism information content (PIC) was calculated by PowerMarker 3.25 software [45].

## 5. Conclusions

A total of 169 gSSR markers were developed from an invasive plant species of *X. strumarium* for the first time, in contrast to the previously limited number of SSR markers. These SSR markers were distributed across all chromosomes, with a distribution of 5–15 markers per chromosome. These 169 gSSR markers can potentially enable marker-assisted taxonomy to resolve ambiguities among morphologically similar *X. strumarium* or *X. sibiricum* species. They can help elucidate population genetic structure and infer invasion routes of *X. strumarium*. Additionally, they can facilitate investigation into the genetic basis of adaptive evolution, particularly by identifying loci under selection associated with invasiveness traits like rapid growth and stress tolerance in future research. In general, they effectively contribute to the molecular identification of taxonomically ambiguous groups and address critical scientific issues such as hybridization, introgression, and adaptive evolution in *Xanthium*.

## Figures and Tables

**Table 1:** Distribution of SSR length (450,847) in the genome of *X. strumarium*.

Length (bp)	Number	Percentage
10–20	221,425	49.11%
21–30	57,037	12.65%
31–40	33,418	7.41%
41–50	21,287	4.72%
51–60	12,557	2.79%
61–70	4858	1.08%
71–80	2034	0.45%
81–90	833	0.18%
91–100	288	0.06%
100+	97,110	21.54%

**Table 2:** (**a**) Distribution of predicted SSR motif types with different numbers (Part 1). (**b**) Distribution of the predicted SSR motif types with different numbers (Part 2).

(**a**)																						
		**No. of Repeats**	**4**		**5**		**6**			**7**		**8**		**9**			**10**		**11**	**12**		**13**
	**No.**																					
**SSR Motif Types**																						
Dinucletides			0		0		36,580			21,928		16,152		11,598			8174		6122	5020		4492
Trinucleotides			88,734		24,411		9725			4213		2087		1334			970		831	770		703
Tetranucleotides			12,482		3011		823			308		157		82			47		35	17		16
Pentanucleotides			3463		713		205			98		71		58			40		34	33		23
Hexanucleotides			2609		620		226			107		60		35			20		19	12		11
Total			107,288		28,755		47,559			26,654		18,527		13,107			9251		7041	5852		5245
Percentage			32.55%		8.72%		14.43%			8.09%		5.62%		3.98%			2.81%		2.14%	1.78%		1.59%
(**b**)																						
		**No. of Repeats**	**14**	**15**		**16**		**17**	**18**		**19**		**20**		**20+**	**Total**		**Percentage**			**Average Physical Distance**	
	**No.**																					
**SSR Motif Types**																						
Dinucletides			4250	4159		4051		3974	3809		3823		3749		26,273	16,4154		49.81%			10.17 kb	
Trinucleotides			641	636		594		531	515		461		454		2212	139,822		42.43%			12.13 kb	
Tetranucleotides			11	5		6		4	3		1		0		5	17,013		5.16%			80.38 kb	
Pentanucleotides			21	10		8		13	9		4		6		7	4816		1.46%			332.88 kb	
Hexanucleotides			9	12		3		2	1		0		2		19	3767		1.14%			442.9 kb	
Total			4932	4822		4662		4524	4337		4289		4211		28,516	329,572		100%			6.91 kb	
Percentage			1.50%	1.46%		1.41%		1.37%	1.32%		1.30%		1.28%		8.65%	100		/			/	

**Table 3:** (**a**) Characteristics and diversity statistics for the 18 SSR markers evaluated in *Xanthium* plants (200 individuals) (Part 1). (**b**) Characteristics and diversity statistics for the 18 SSR markers evaluated in *Xanthium* plants (200 individuals) (Part 2).

(**a**)																
	**Locus**	**INVZ-191**	**INVZ-456**	**INVZ-705**	**INVZ-864**	**INVZ-1104**		**INVZ-1274**		**INVZ-1501**		**INVZ-1652**		**INVZ-1925**		**INVZ-2051**
**Types**																
No. of Different Alleles (Na)		1.200 ± 0.302	1.700 ± 0.483	2.400 ± 0.369	2.900 ± 0.920	1.500 ± 0.506		3.200 ± 1.207		2.400 ± 0.769		2.400 ± 0.500		2.100 ± 1.282		1.500 ± 0.608
No. of Effective Alleles (Ne)		1.066 ± 0.120	1.282 ± 0.303	2.185 ± 0.403	2.395 ± 0.460	1.350 ± 0.351		2.261 ± 0.341		1.933 ± 0.326		2.101 ± 0.272		1.992 ± 1.164		1.465 ± 0.596
Shannon’s Information Index (I)		0.076 ± 0.056	0.250 ± 0.225	0.767 ± 0.196	0.890 ± 0.212	0.265 ± 0.252		0.885 ± 0.193		0.669 ± 0.210		0.763 ± 0.136		0.702 ± 0.414		0.474 ± 0.235
Observed Heterozygosity (Ho)		0.000 ± 0.001	0.242 ± 0.128	0.800 ± 0.221	1.000 ± 0.001	0.326 ± 0.146		0.950 ± 0.113		0.816 ± 0.261		0.924 ± 0.139		0.700 ± 0.346		0.590 ± 0.364
Expected Heterozygosity (He)		0.046 ± 0.035	0.156 ± 0.154	0.507 ± 0.119	0.561 ± 0.065	0.181 ± 0.174		0.544 ± 0.054		0.445 ± 0.132		0.510 ± 0.063		0.424 ± 0.223		0.340 ± 0.169
Polymorphism Information Content (PIC)		0.215 ± 0.117	0.343 ± 0.115	0.801 ± 0.066	0.778 ± 0.078	0.839 ± 0.138		0.853 ± 0.045		0.612 ± 0.148		0.691 ± 0.065		0.852 ± 0.195		0.774 ± 0.162
(**b**)																
	**Locus**	**INVZ-2330**	**INVZ-2478**		**INVZ-2645**		**INVZ-2906**		**INVZ-3183**		**INVZ-3458**		**INVZ-3563**		**INVZ-3764**	**Average**
**Types**																
No. of Different Alleles (Na)		1.200 ± 0.812	1.8 ± 0.452		2.300 ± 0.483		2.000 ± 0.001		1.200 ± 0.739		2.600 ± 1.129		1.700 ± 0.830		2.000 ± 0.001	2.006
No. of Effective Alleles (Ne)		1.077 ± 0.744	1.8 ± 0.452		2.090 ± 0.202		2.000 ± 0.001		1.200 ± 0.739		1.851 ± 0.557		1.104 ± 0.328		2.000 ± 0.001	1.730
Shannon’s Information Index (I)		0.332 ± 0.288	0.624 ± 0.157		0.756 ± 0.116		0.693 ± 0.001		0.416 ± 0.256		0.712 ± 0.220		0.239 ± 0.202		0.693 ± 0.001	0.567
Observed Heterozygosity (Ho)		0.410 ± 0.364	0.900 ± 0.226		0.945 ± 0.112		1.000 ± 0.001		0.600 ± 0.370		0.714 ± 0.385		0.170 ± 0.151		1.000 ± 0.001	0.672
Expected Heterozygosity (He)		0.222 ± 0.193	0.450 ± 0.113		0.515 ± 0.042		0.500 ± 0.001		0.300 ± 0.185		0.448 ± 0.127		0.138 ± 0.120		0.500 ± 0.001	0.377
Polymorphism Information Content (PIC)		0.678 ± 0.170	0.491 ± 0.158		0.623 ± 0.063		0.644 ± 0.043		0.375 ± 0.203		0.812 ± 0.153		0.161 ± 0.090		0.614 ± 0.053	0.620

## Data Availability

The data presented in this study are available in the text and Supplementary Materials. The data presented in this study are available upon request from the corresponding author.

## Supplementary Materials

The following supporting information can be downloaded at: https://www.mdpi.com/article/10.3390/plants14223522/s1, Figure S1. Number of predicted SSRs with different chromosome in *X. strumarium*. The *x*-axis represents Chromosome, and the *y*-axis represents Number of predicted SSRs. The line chart displays the fluctuating trend of predicted SSR numbers across different chromosomes. Figure S2. The 169 SSR markers with different chromosomes in *X. strumarium*. Chr + a number represents different chromosomes. Xst and Xsi represent *Xanthium strumarium* and *Xanthium sibiricum*, respectively. Table S1. The number and proportion of SSRs on different chromosomes of *X. strumarium*. Number represents the total count of SSRs identified on each specific chromosome, and Percentage indicates the proportion of SSRs on that chromosome relative to the total SSRs. Table S2. 169 SSR loci information of *X. strumarium*. SSR motif: The repeating nucleotide sequence (e.g., TG, GA, AT) that defines the SSR. SSR length: The total length of the SSR sequence. SSR repeat number: The number of times the SSR motif is repeated within the SSR sequence. Location: The genomic position of the SSR locus. Sequence: The DNA sequence containing the SSR motif. SSR Name: A unique identifier for each SSR locus. Left primer/Right primer: The forward and reverse PCR primers designed for amplifying the SSR locus. SSR amplicon size: The expected size of the PCR product when the locus is amplified using the specified primers. Table S3. Distribution of the verified 169 SSR motif types with different numbers. Rows represent specific SSR motifs (e.g., AT, TA, AG), and columns indicate the number of repeats (ranging from 4 to 27). Values in the table represent the count of each SSR motif type with a given repeat number.
