# Supplementary material for: Genome-Wide Development and Characterization of 169 gSSR Markers in the Invasive Plant Xanthium strumarium L
Source: Plants (Basel). 2025 Nov 18;14(22):3522. doi: 10.3390/plants14223522 (PMC12656314; doi:10.3390/plants14223522)
Supplement: Supplementary file 1 [file plants-14-03522-s001.zip › Fig. S2.pptx]

## Slide 1
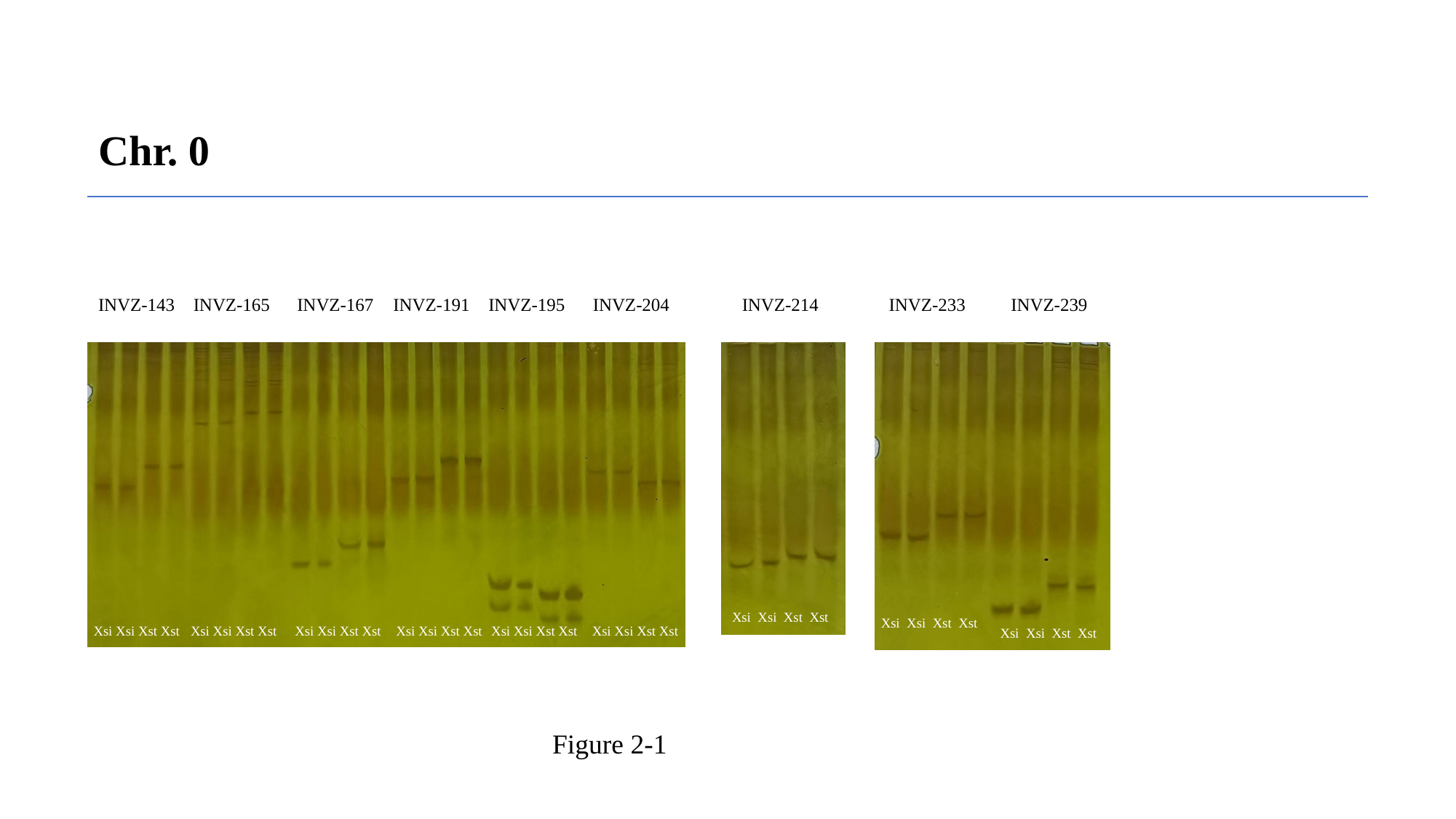

Chr. 0
INVZ-143
INVZ-165
INVZ-167
INVZ-191
INVZ-195
INVZ-204
INVZ-214
INVZ-233
INVZ-239
Xsi Xsi Xst Xst
Xsi Xsi Xst Xst
Xsi Xsi Xst Xst
Xsi Xsi Xst Xst
Xsi Xsi Xst Xst
Xsi Xsi Xst Xst
Xsi Xsi Xst Xst
Xsi Xsi Xst Xst
Xsi Xsi Xst Xst
Figure 2-1

## Slide 2
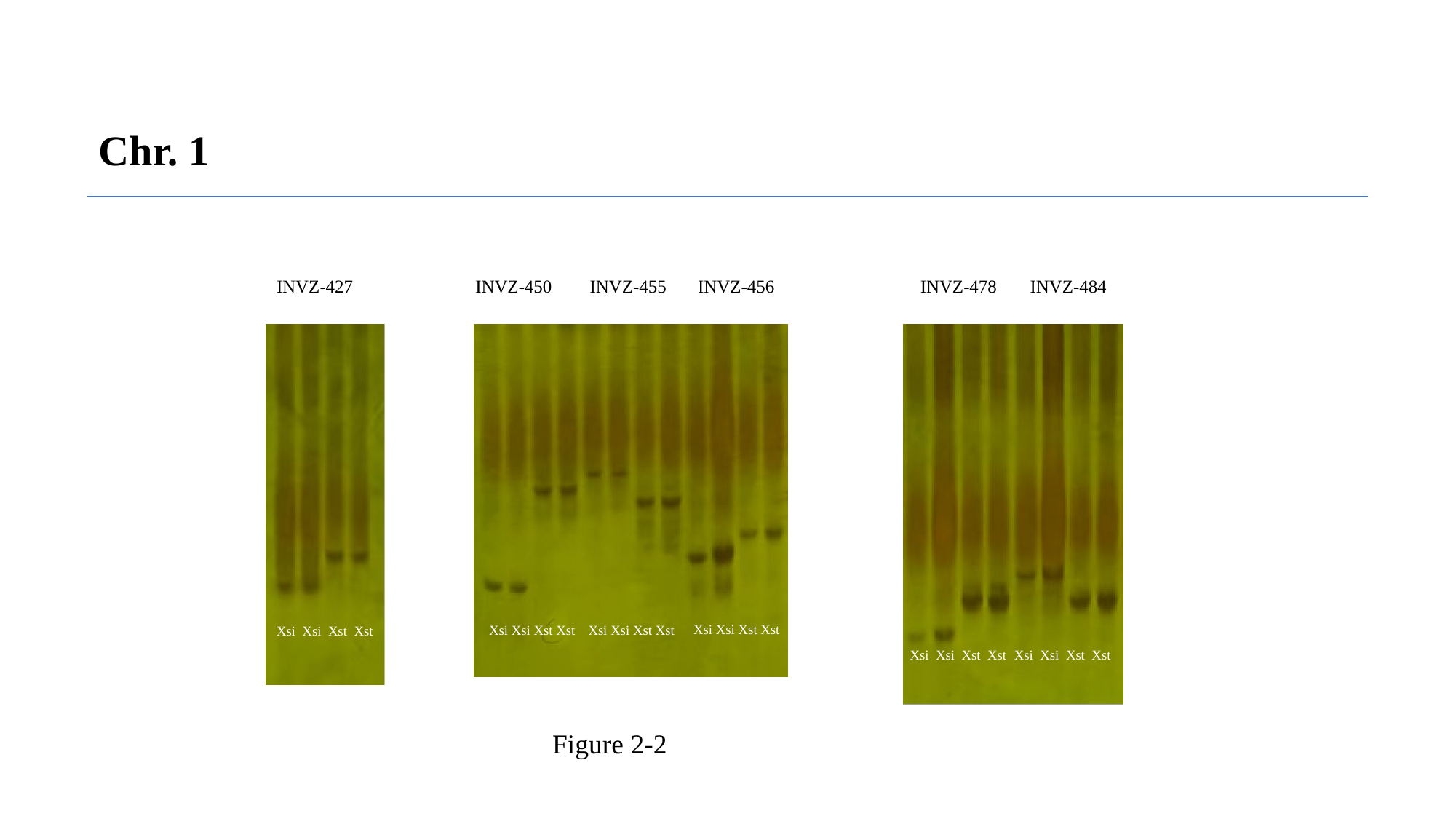

Chr. 1
INVZ-427
INVZ-450
INVZ-455
INVZ-456
INVZ-478
INVZ-484
Xsi Xsi Xst Xst
Xsi Xsi Xst Xst
Xsi Xsi Xst Xst
Xsi Xsi Xst Xst
Xsi Xsi Xst Xst
Xsi Xsi Xst Xst
Figure 2-2

## Slide 3
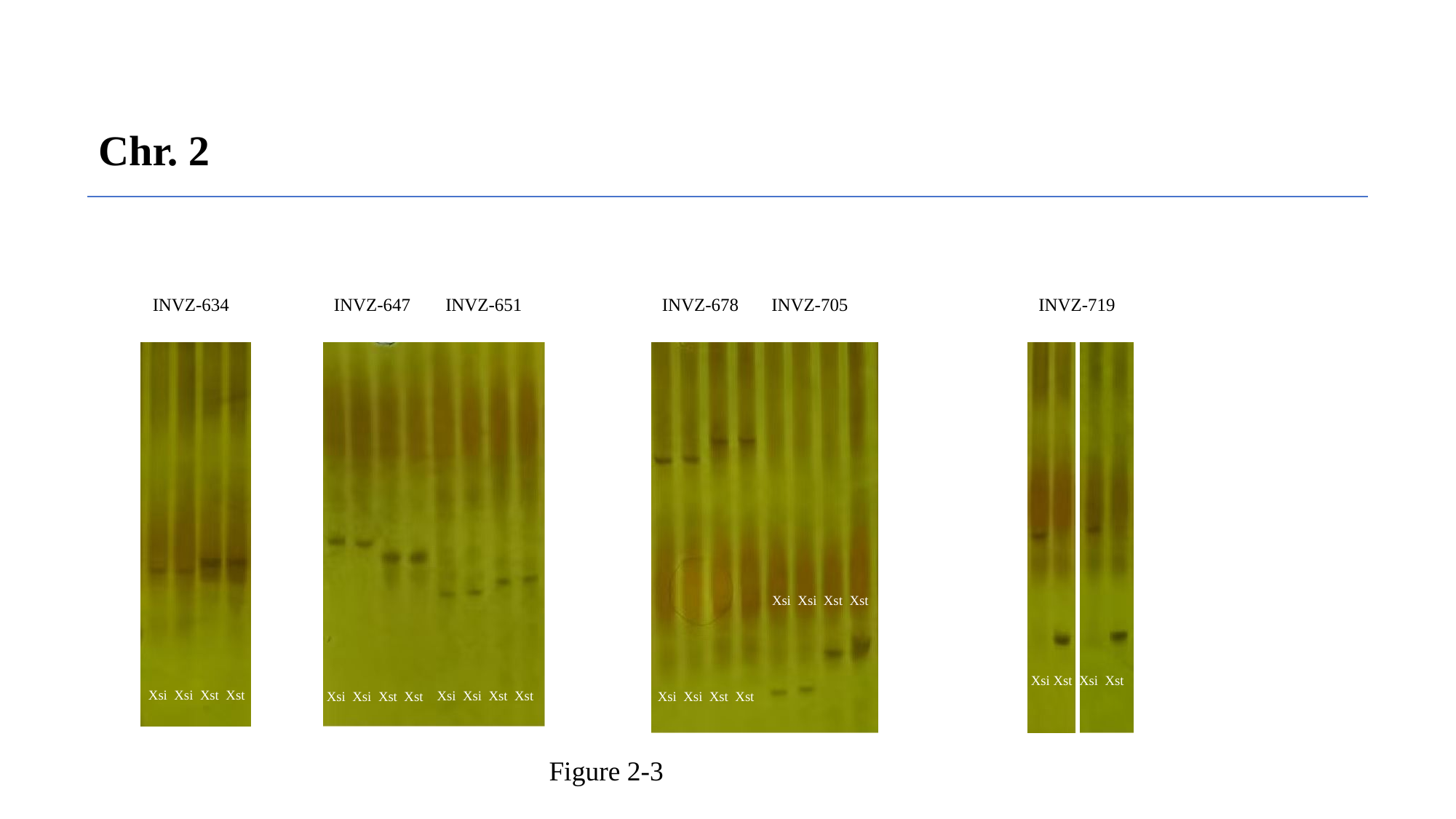

Chr. 2
INVZ-634
INVZ-678
INVZ-719
INVZ-647
INVZ-651
INVZ-705
Xsi Xsi Xst Xst
Xsi Xst Xsi Xst
Xsi Xsi Xst Xst
Xsi Xsi Xst Xst
Xsi Xsi Xst Xst
Xsi Xsi Xst Xst
Figure 2-3

## Slide 4
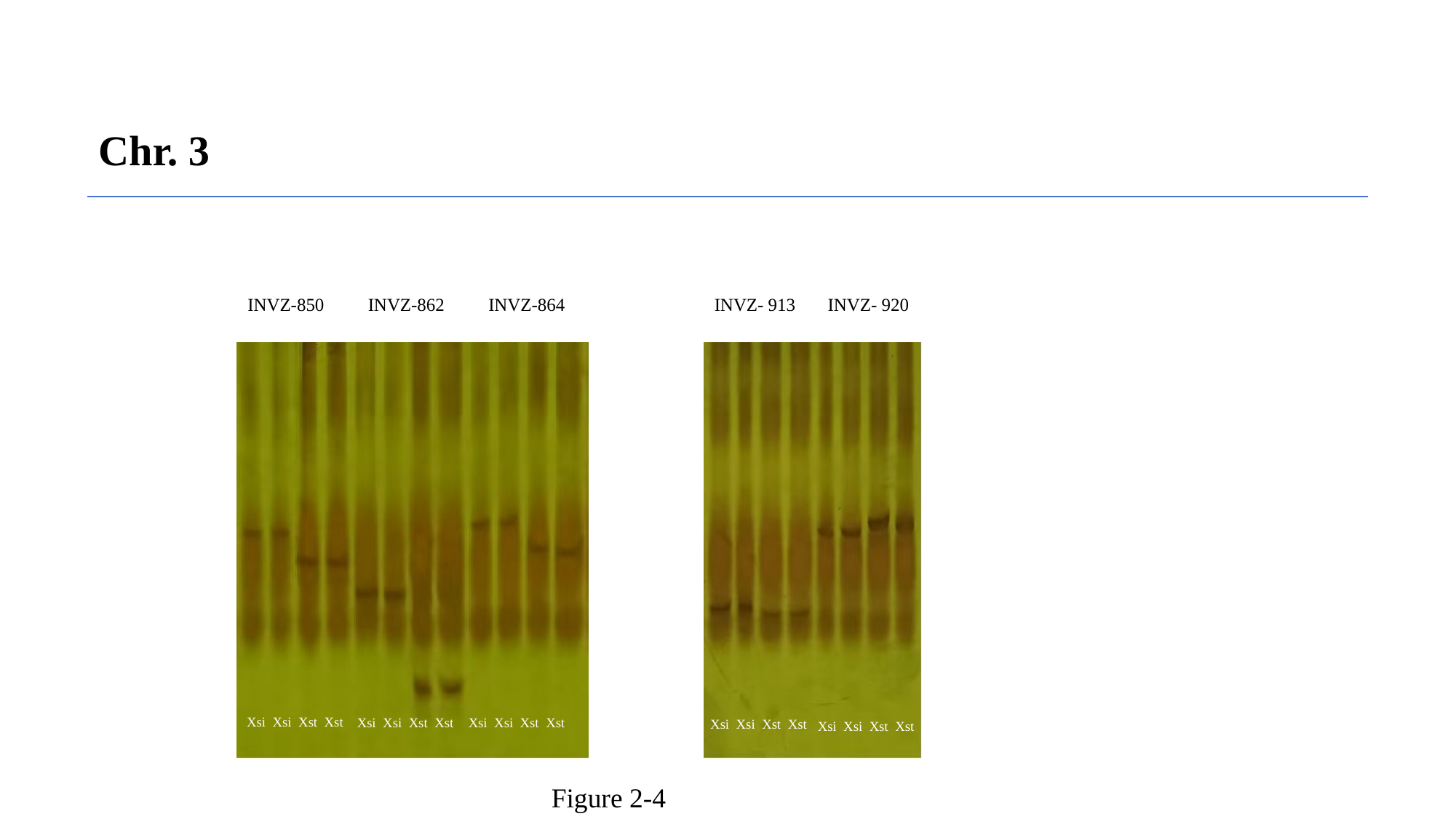

Chr. 3
INVZ-850
INVZ-862
INVZ-864
INVZ- 913
INVZ- 920
Xsi Xsi Xst Xst
Xsi Xsi Xst Xst
Xsi Xsi Xst Xst
Xsi Xsi Xst Xst
Xsi Xsi Xst Xst
Figure 2-4

## Slide 5
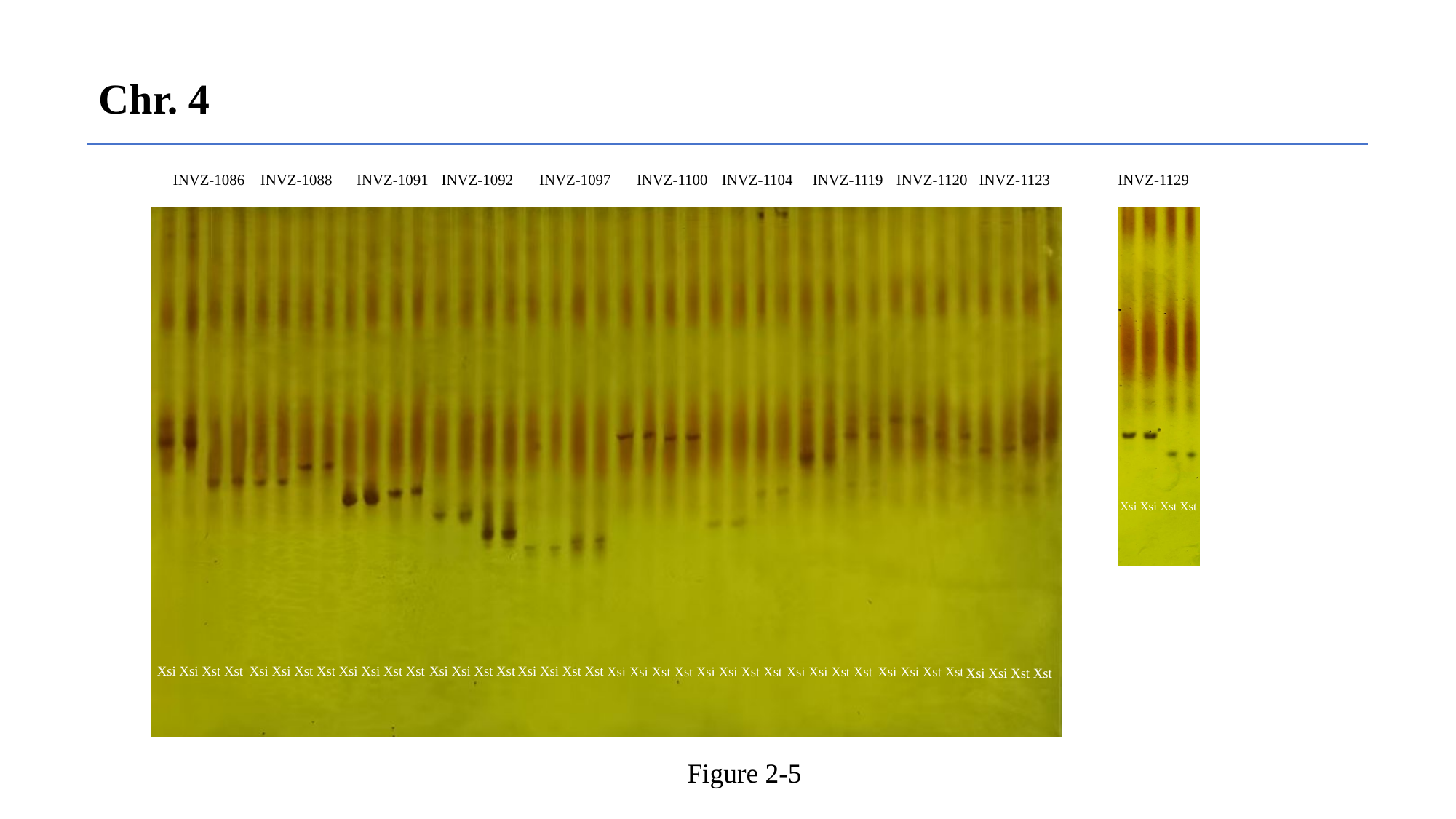

Chr. 4
INVZ-1086
INVZ-1088
INVZ-1091
INVZ-1092
INVZ-1097
INVZ-1100
INVZ-1104
INVZ-1119
INVZ-1120
INVZ-1123
INVZ-1129
Xsi Xsi Xst Xst
Xsi Xsi Xst Xst
Xsi Xsi Xst Xst
Xsi Xsi Xst Xst
Xsi Xsi Xst Xst
Xsi Xsi Xst Xst
Xsi Xsi Xst Xst
Xsi Xsi Xst Xst
Xsi Xsi Xst Xst
Xsi Xsi Xst Xst
Xsi Xsi Xst Xst
Figure 2-5

## Slide 6
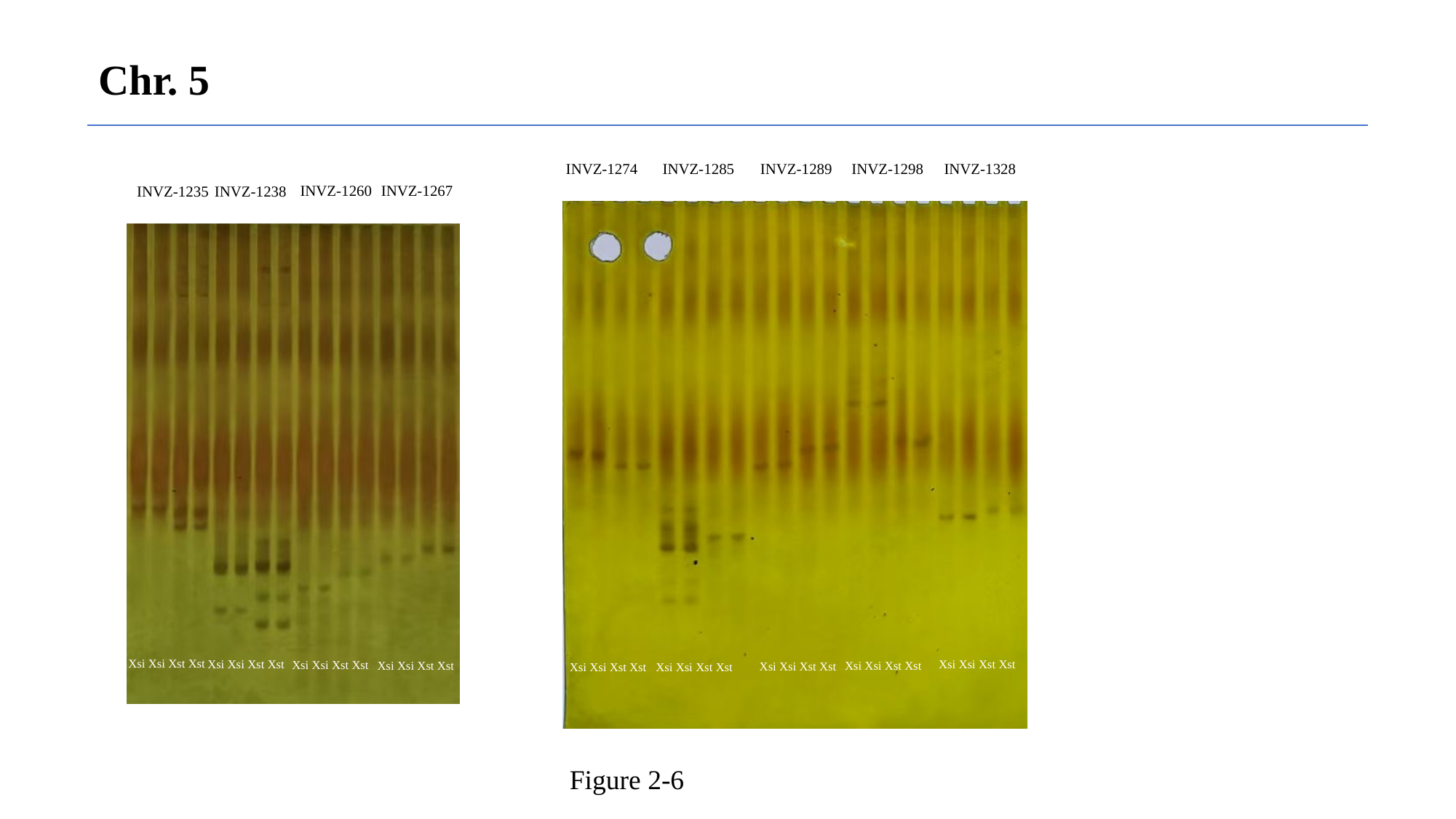

Chr. 5
INVZ-1274
INVZ-1285
INVZ-1289
INVZ-1298
INVZ-1328
INVZ-1260
INVZ-1267
INVZ-1235
INVZ-1238
Xsi Xsi Xst Xst
Xsi Xsi Xst Xst
Xsi Xsi Xst Xst
Xsi Xsi Xst Xst
Xsi Xsi Xst Xst
Xsi Xsi Xst Xst
Xsi Xsi Xst Xst
Xsi Xsi Xst Xst
Xsi Xsi Xst Xst
Figure 2-6

## Slide 7
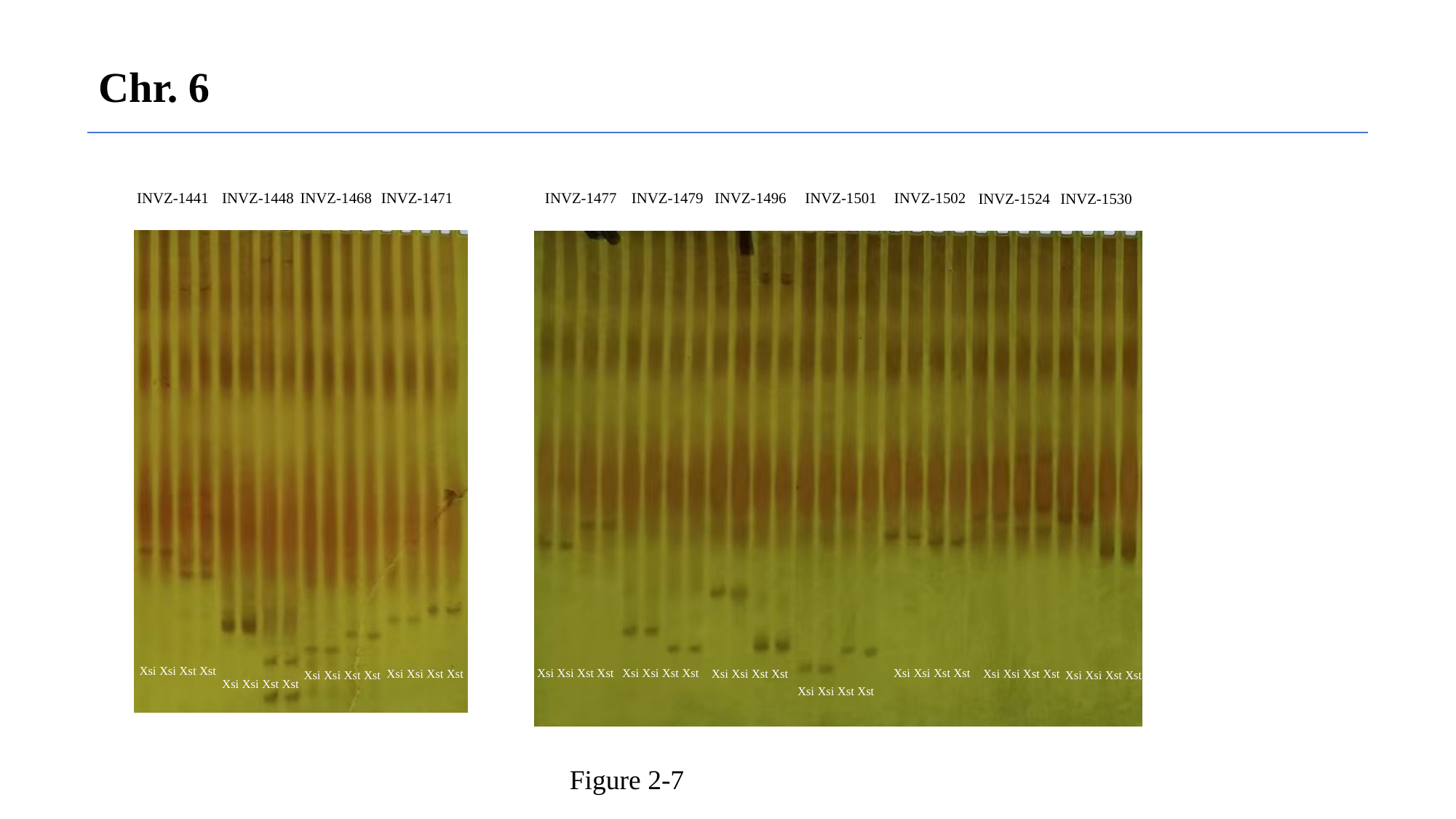

Chr. 6
INVZ-1468
INVZ-1477
INVZ-1496
INVZ-1441
INVZ-1448
INVZ-1471
INVZ-1501
INVZ-1502
INVZ-1479
INVZ-1524
INVZ-1530
Xsi Xsi Xst Xst
Xsi Xsi Xst Xst
Xsi Xsi Xst Xst
Xsi Xsi Xst Xst
Xsi Xsi Xst Xst
Xsi Xsi Xst Xst
Xsi Xsi Xst Xst
Xsi Xsi Xst Xst
Xsi Xsi Xst Xst
Xsi Xsi Xst Xst
Xsi Xsi Xst Xst
Figure 2-7

## Slide 8
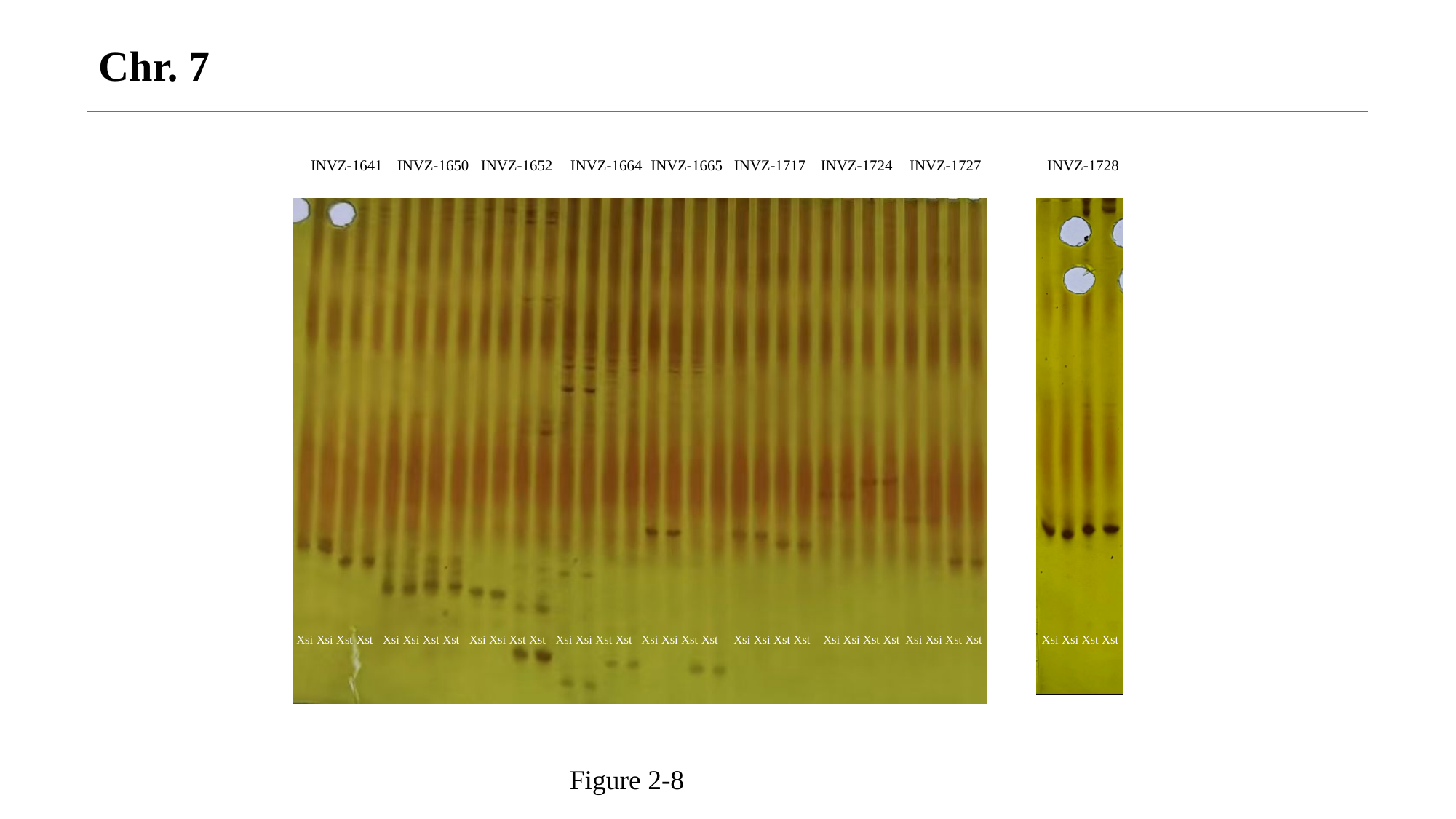

Chr. 7
INVZ-1641
INVZ-1650
INVZ-1652
INVZ-1664
INVZ-1665
INVZ-1717
INVZ-1724
INVZ-1727
INVZ-1728
Xsi Xsi Xst Xst
Xsi Xsi Xst Xst
Xsi Xsi Xst Xst
Xsi Xsi Xst Xst
Xsi Xsi Xst Xst
Xsi Xsi Xst Xst
Xsi Xsi Xst Xst
Xsi Xsi Xst Xst
Xsi Xsi Xst Xst
Figure 2-8

## Slide 9
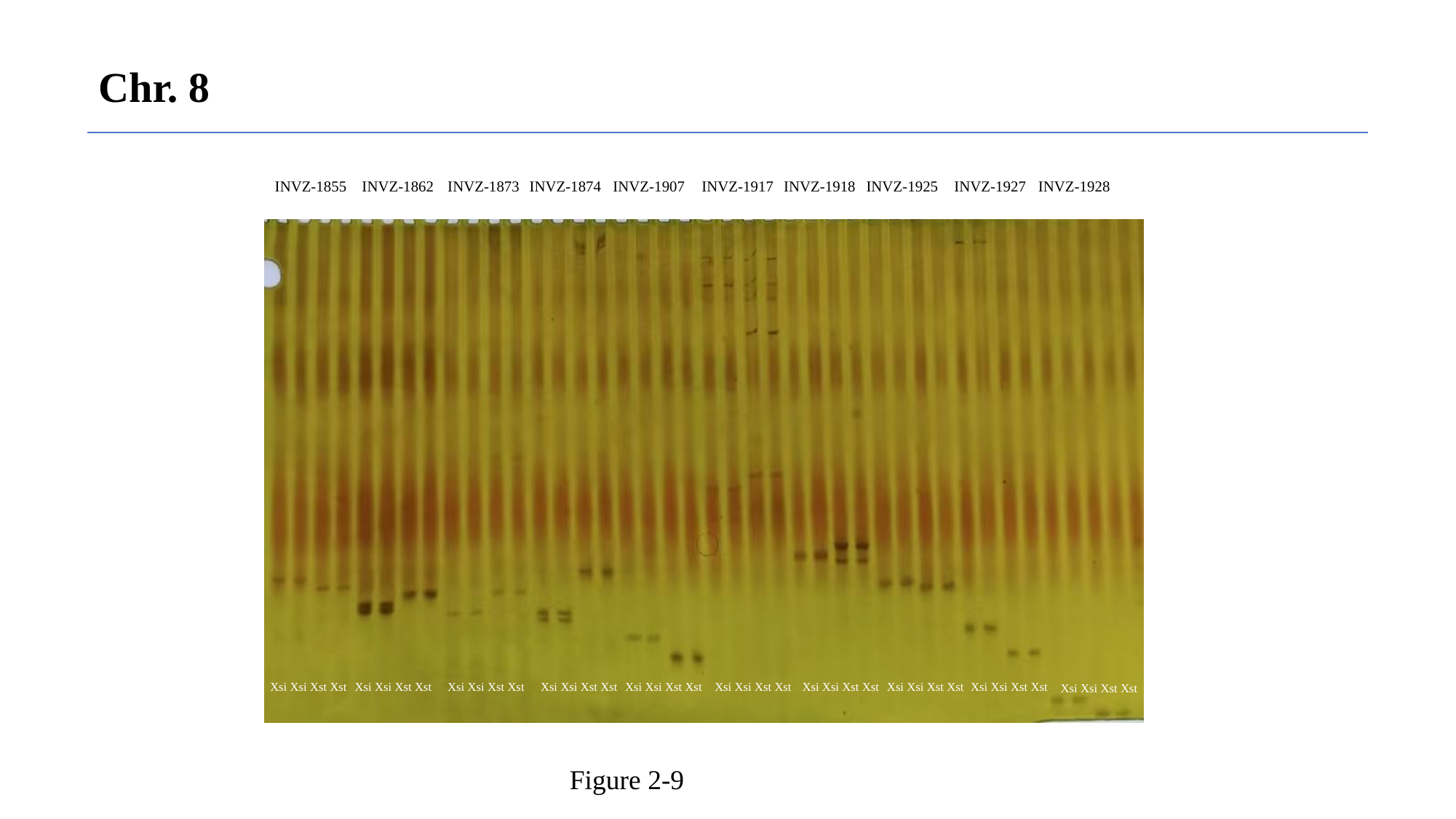

Chr. 8
INVZ-1855
INVZ-1862
INVZ-1873
INVZ-1874
INVZ-1907
INVZ-1917
INVZ-1918
INVZ-1925
INVZ-1927
INVZ-1928
Xsi Xsi Xst Xst
Xsi Xsi Xst Xst
Xsi Xsi Xst Xst
Xsi Xsi Xst Xst
Xsi Xsi Xst Xst
Xsi Xsi Xst Xst
Xsi Xsi Xst Xst
Xsi Xsi Xst Xst
Xsi Xsi Xst Xst
Xsi Xsi Xst Xst
Figure 2-9

## Slide 10
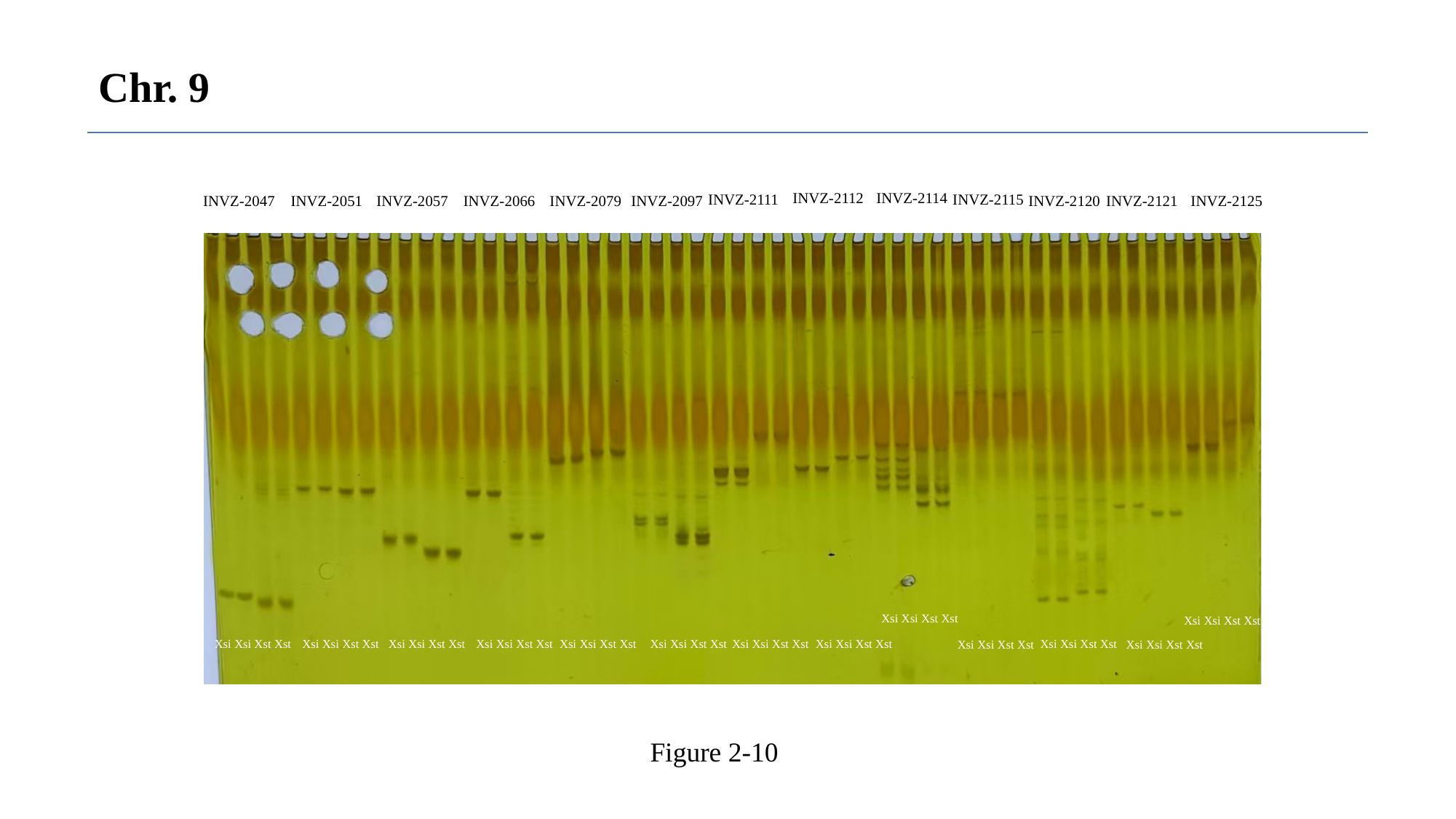

Chr. 9
INVZ-2112
INVZ-2114
INVZ-2111
INVZ-2115
INVZ-2047
INVZ-2051
INVZ-2057
INVZ-2066
INVZ-2079
INVZ-2097
INVZ-2120
INVZ-2121
INVZ-2125
Xsi Xsi Xst Xst
Xsi Xsi Xst Xst
Xsi Xsi Xst Xst
Xsi Xsi Xst Xst
Xsi Xsi Xst Xst
Xsi Xsi Xst Xst
Xsi Xsi Xst Xst
Xsi Xsi Xst Xst
Xsi Xsi Xst Xst
Xsi Xsi Xst Xst
Xsi Xsi Xst Xst
Xsi Xsi Xst Xst
Xsi Xsi Xst Xst
Figure 2-10

## Slide 11
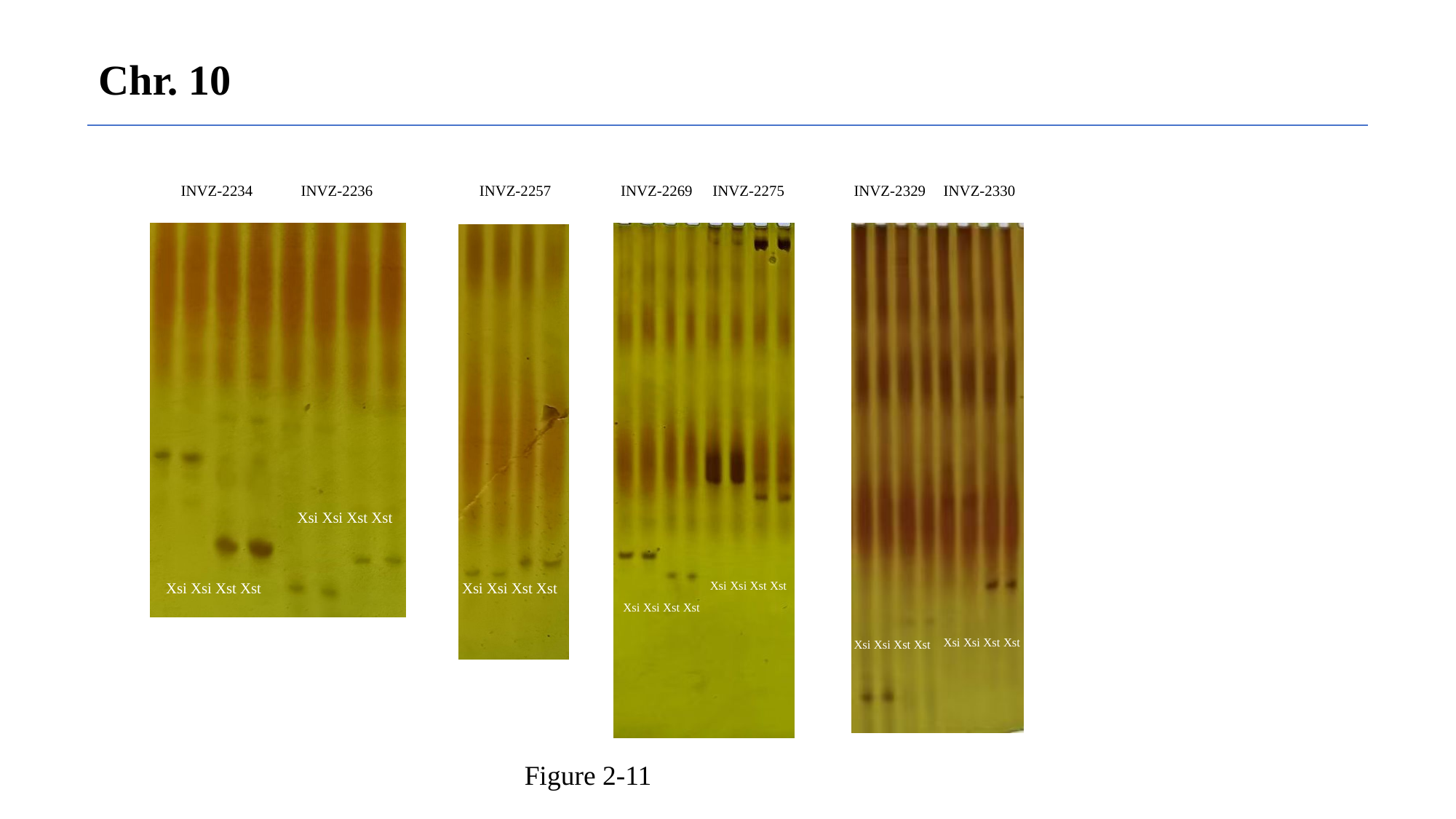

Chr. 10
INVZ-2234
INVZ-2236
INVZ-2257
INVZ-2269
INVZ-2275
INVZ-2329
INVZ-2330
Xsi Xsi Xst Xst
Xsi Xsi Xst Xst
Xsi Xsi Xst Xst
Xsi Xsi Xst Xst
Xsi Xsi Xst Xst
Xsi Xsi Xst Xst
Xsi Xsi Xst Xst
Figure 2-11

## Slide 12
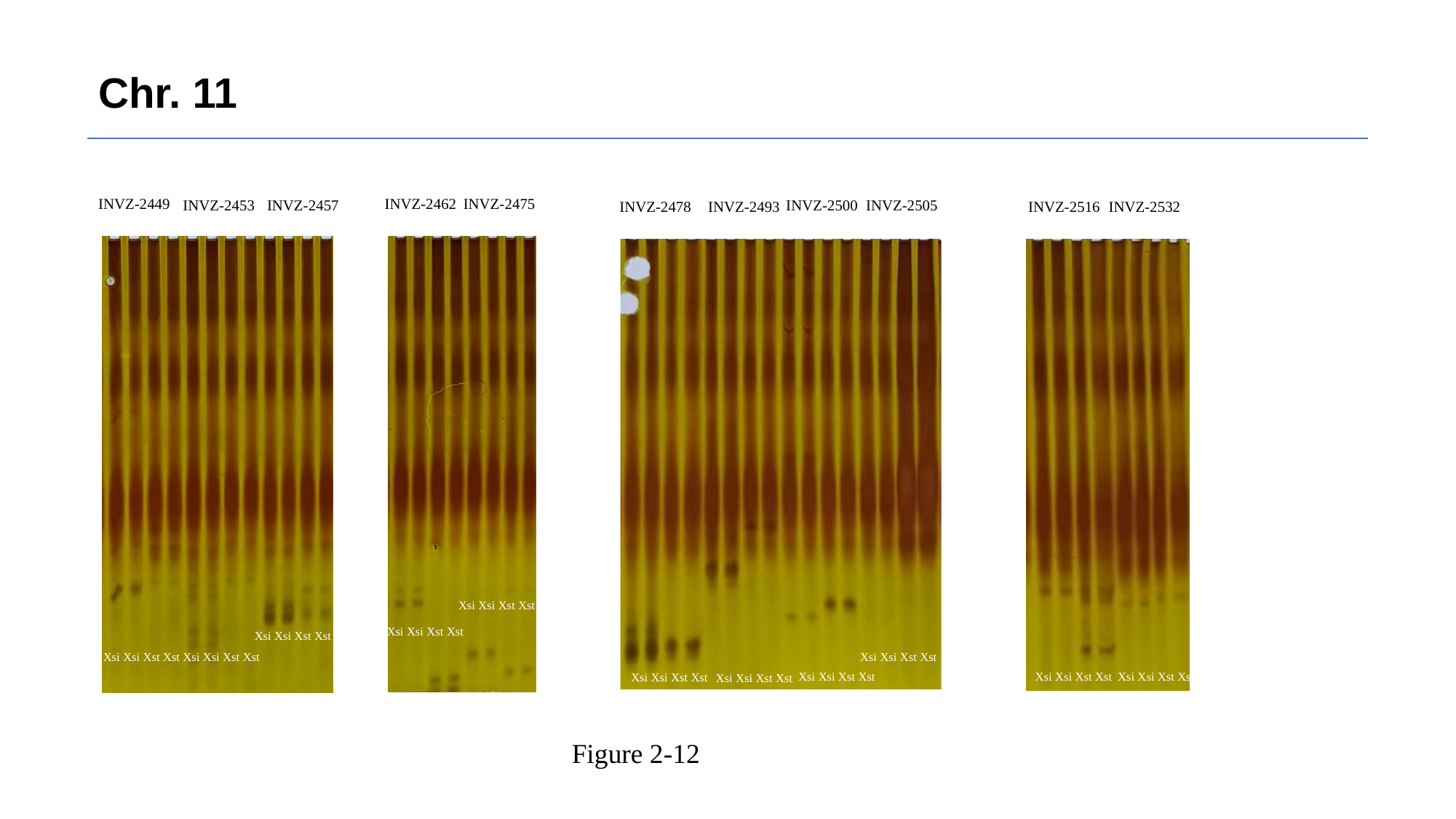

Chr. 11
INVZ-2449
INVZ-2462
INVZ-2475
INVZ-2453
INVZ-2457
INVZ-2505
INVZ-2500
INVZ-2532
INVZ-2478
INVZ-2493
INVZ-2516
Xsi Xsi Xst Xst
Xsi Xsi Xst Xst
Xsi Xsi Xst Xst
Xsi Xsi Xst Xst
Xsi Xsi Xst Xst
Xsi Xsi Xst Xst
Xsi Xsi Xst Xst
Xsi Xsi Xst Xst
Xsi Xsi Xst Xst
Xsi Xsi Xst Xst
Xsi Xsi Xst Xst
Figure 2-12

## Slide 13
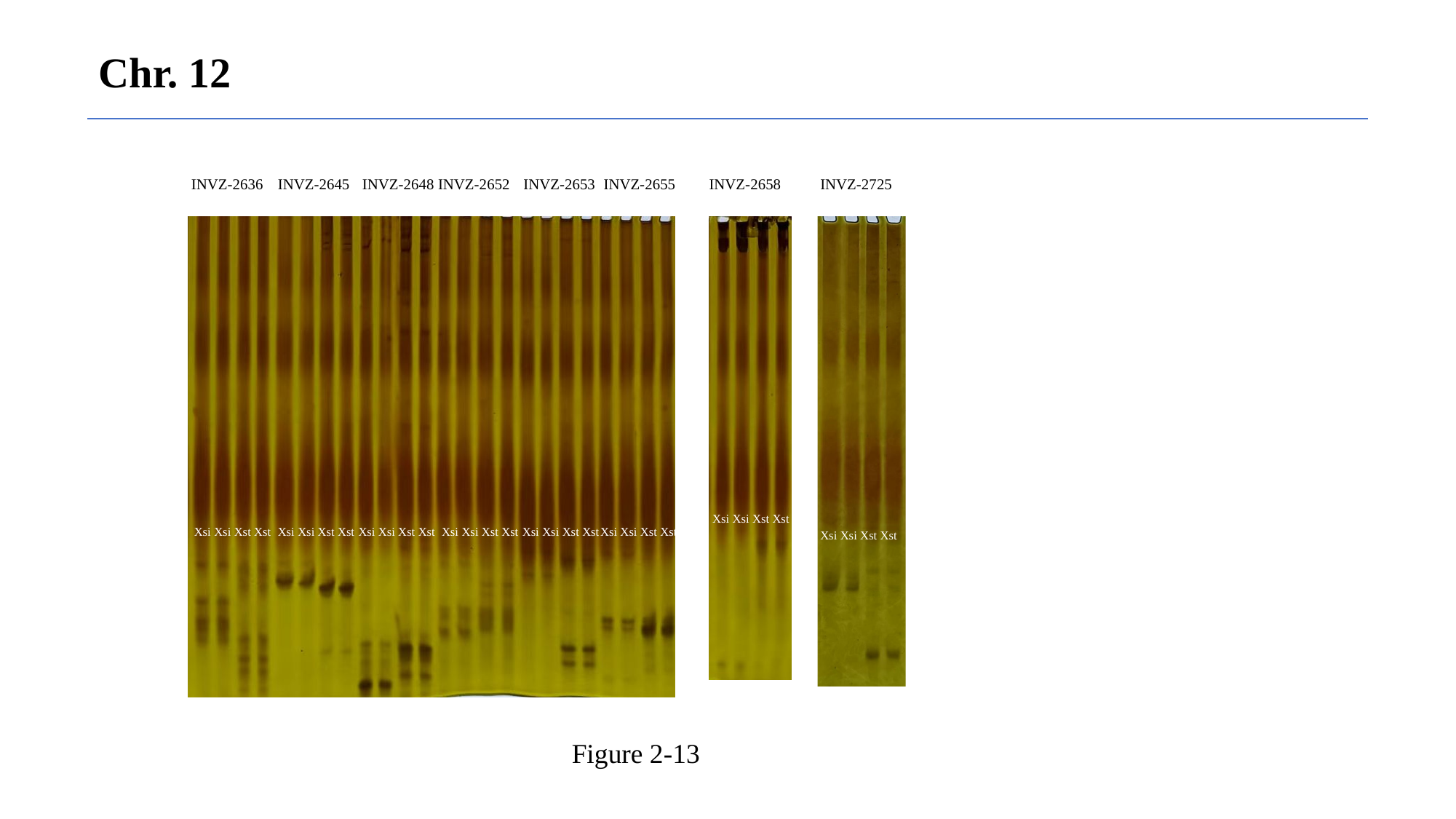

Chr. 12
INVZ-2636
INVZ-2645
INVZ-2648
INVZ-2652
INVZ-2653
INVZ-2655
INVZ-2658
INVZ-2725
Xsi Xsi Xst Xst
Xsi Xsi Xst Xst
Xsi Xsi Xst Xst
Xsi Xsi Xst Xst
Xsi Xsi Xst Xst
Xsi Xsi Xst Xst
Xsi Xsi Xst Xst
Xsi Xsi Xst Xst
Figure 2-13

## Slide 14
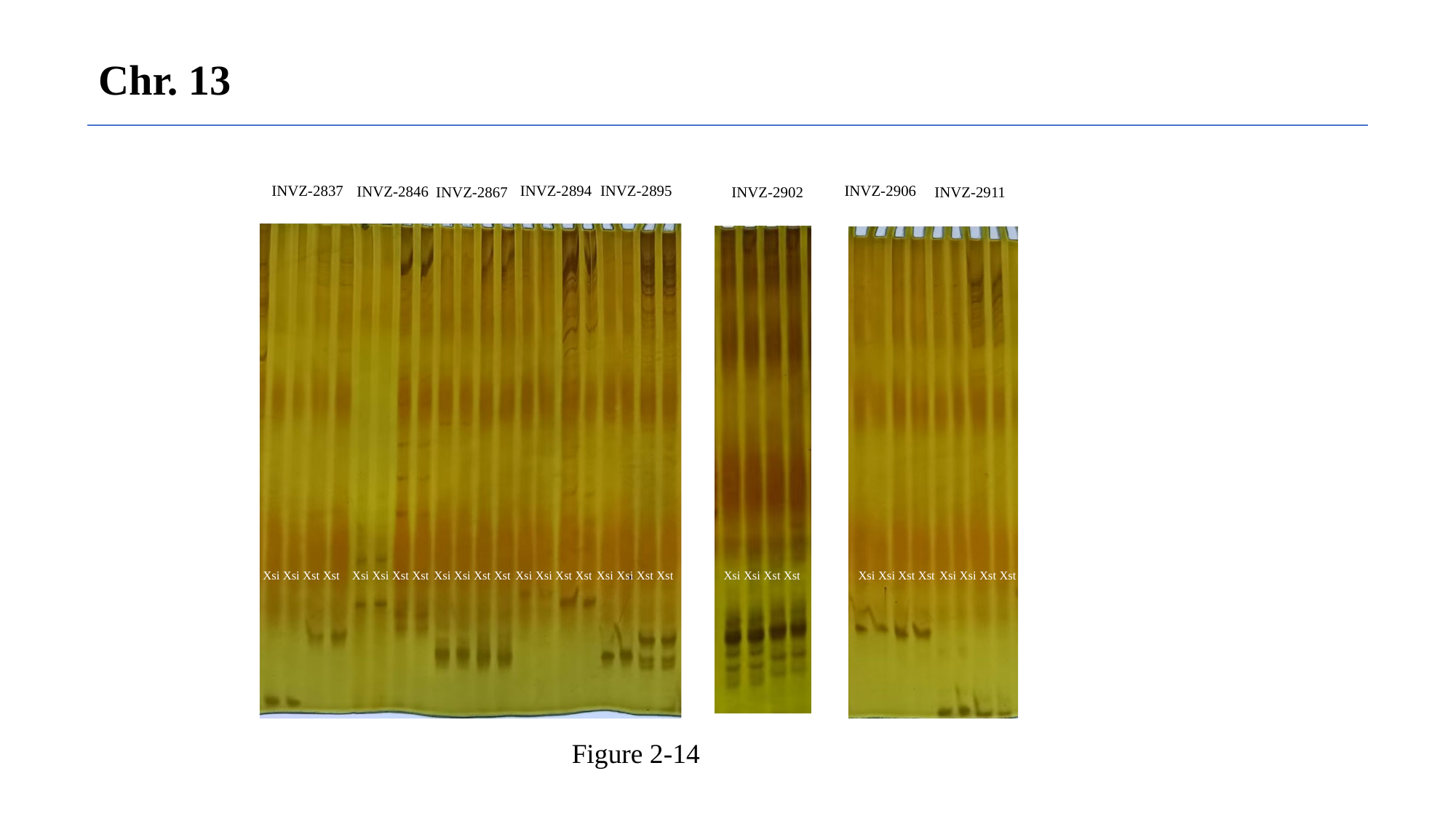

Chr. 13
INVZ-2837
INVZ-2894
INVZ-2895
INVZ-2906
INVZ-2846
INVZ-2867
INVZ-2911
INVZ-2902
Xsi Xsi Xst Xst
Xsi Xsi Xst Xst
Xsi Xsi Xst Xst
Xsi Xsi Xst Xst
Xsi Xsi Xst Xst
Xsi Xsi Xst Xst
Xsi Xsi Xst Xst
Xsi Xsi Xst Xst
Figure 2-14

## Slide 15
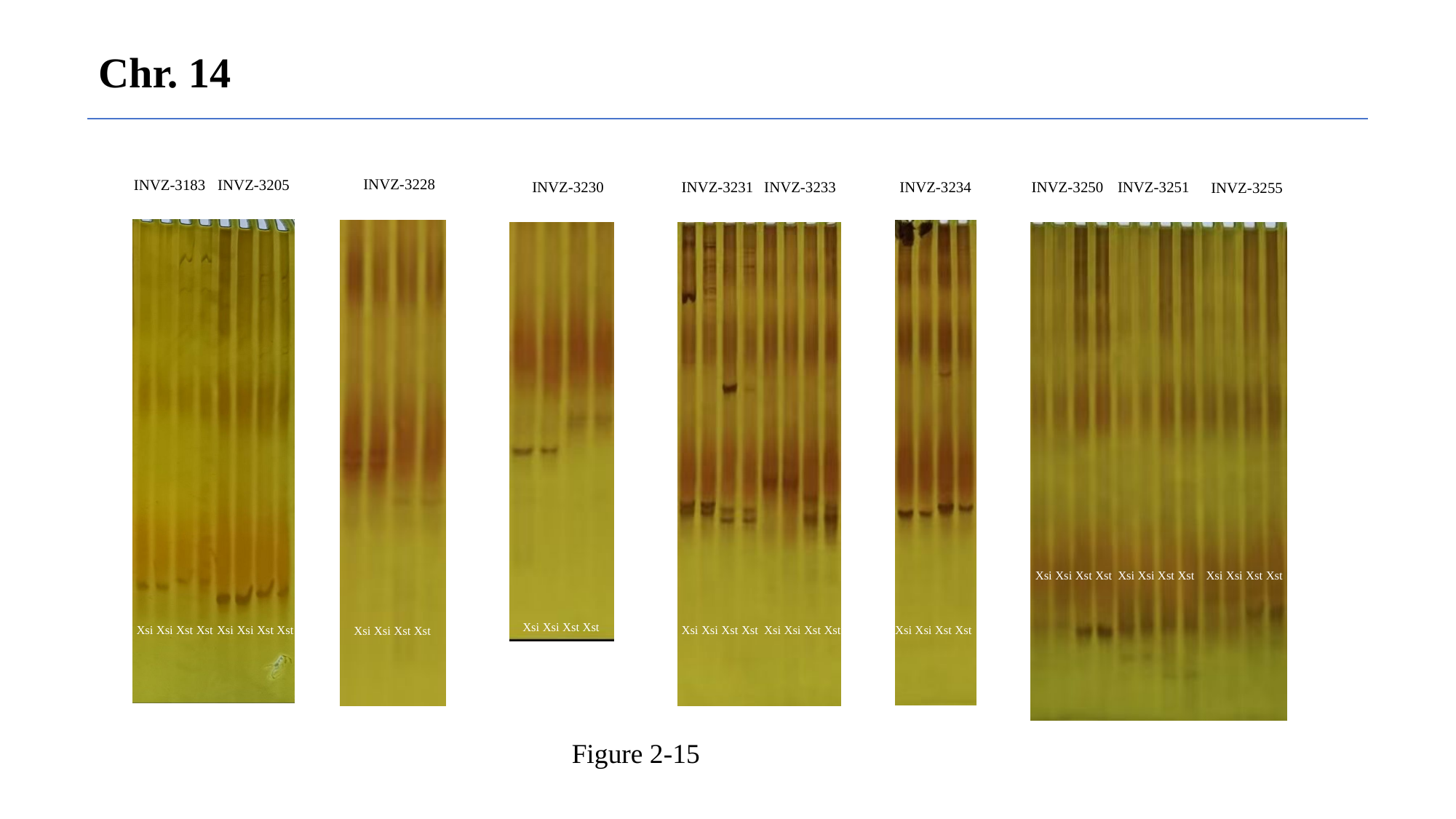

Chr. 14
INVZ-3228
INVZ-3183
INVZ-3205
INVZ-3233
INVZ-3230
INVZ-3231
INVZ-3250
INVZ-3251
INVZ-3234
INVZ-3255
Xsi Xsi Xst Xst
Xsi Xsi Xst Xst
Xsi Xsi Xst Xst
Xsi Xsi Xst Xst
Xsi Xsi Xst Xst
Xsi Xsi Xst Xst
Xsi Xsi Xst Xst
Xsi Xsi Xst Xst
Xsi Xsi Xst Xst
Xsi Xsi Xst Xst
Figure 2-15

## Slide 16
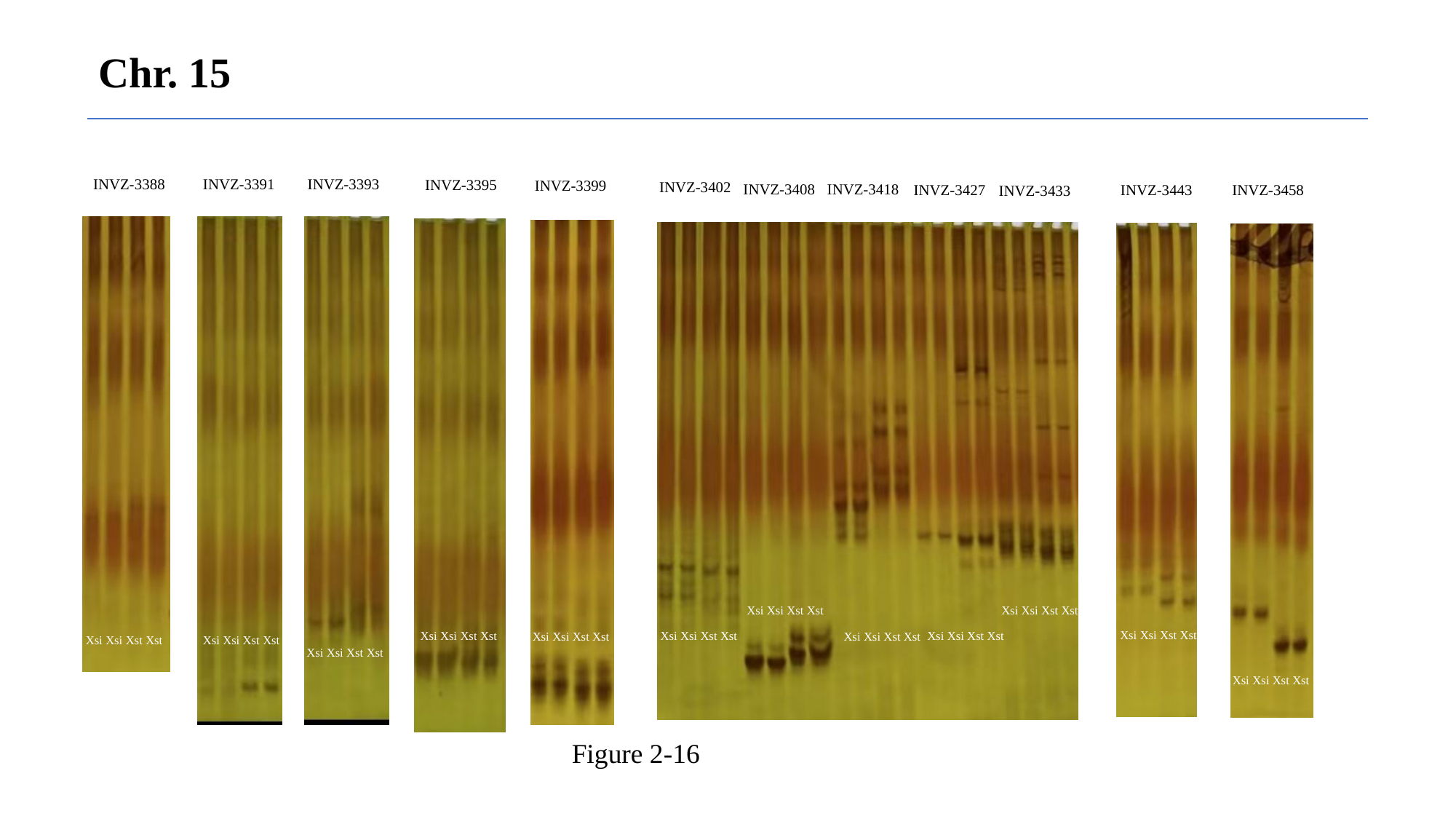

Chr. 15
INVZ-3388
INVZ-3391
INVZ-3393
INVZ-3395
INVZ-3399
INVZ-3402
INVZ-3418
INVZ-3408
INVZ-3427
INVZ-3443
INVZ-3458
INVZ-3433
Xsi Xsi Xst Xst
Xsi Xsi Xst Xst
Xsi Xsi Xst Xst
Xsi Xsi Xst Xst
Xsi Xsi Xst Xst
Xsi Xsi Xst Xst
Xsi Xsi Xst Xst
Xsi Xsi Xst Xst
Xsi Xsi Xst Xst
Xsi Xsi Xst Xst
Xsi Xsi Xst Xst
Xsi Xsi Xst Xst
Figure 2-16

## Slide 17
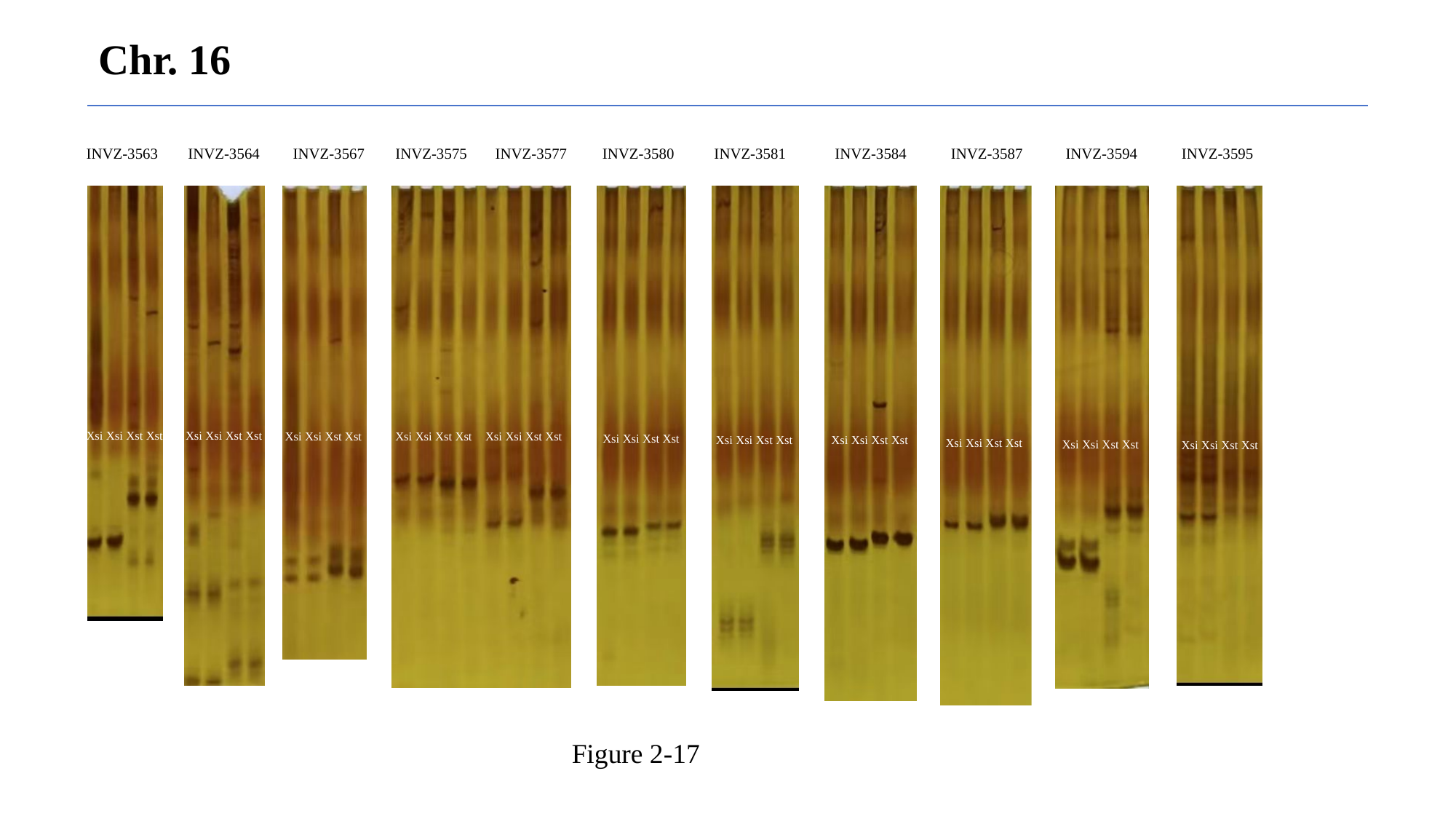

Chr. 16
INVZ-3563
INVZ-3564
INVZ-3567
INVZ-3575
INVZ-3577
INVZ-3580
INVZ-3581
INVZ-3584
INVZ-3587
INVZ-3594
INVZ-3595
Xsi Xsi Xst Xst
Xsi Xsi Xst Xst
Xsi Xsi Xst Xst
Xsi Xsi Xst Xst
Xsi Xsi Xst Xst
Xsi Xsi Xst Xst
Xsi Xsi Xst Xst
Xsi Xsi Xst Xst
Xsi Xsi Xst Xst
Xsi Xsi Xst Xst
Xsi Xsi Xst Xst
Figure 2-17

## Slide 18
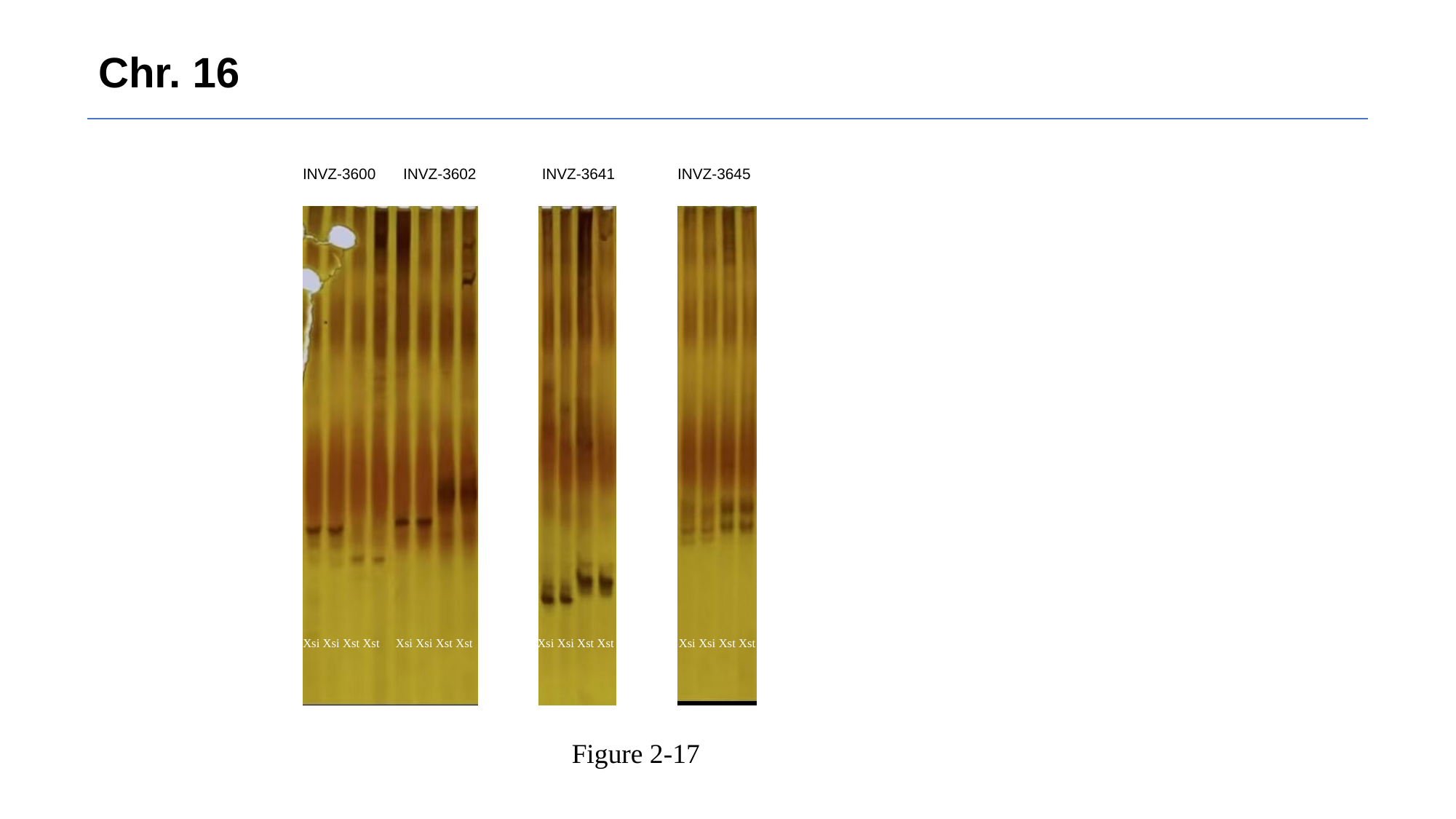

Chr. 16
INVZ-3600
INVZ-3602
INVZ-3641
INVZ-3645
Xsi Xsi Xst Xst
Xsi Xsi Xst Xst
Xsi Xsi Xst Xst
Xsi Xsi Xst Xst
Figure 2-17

## Slide 19
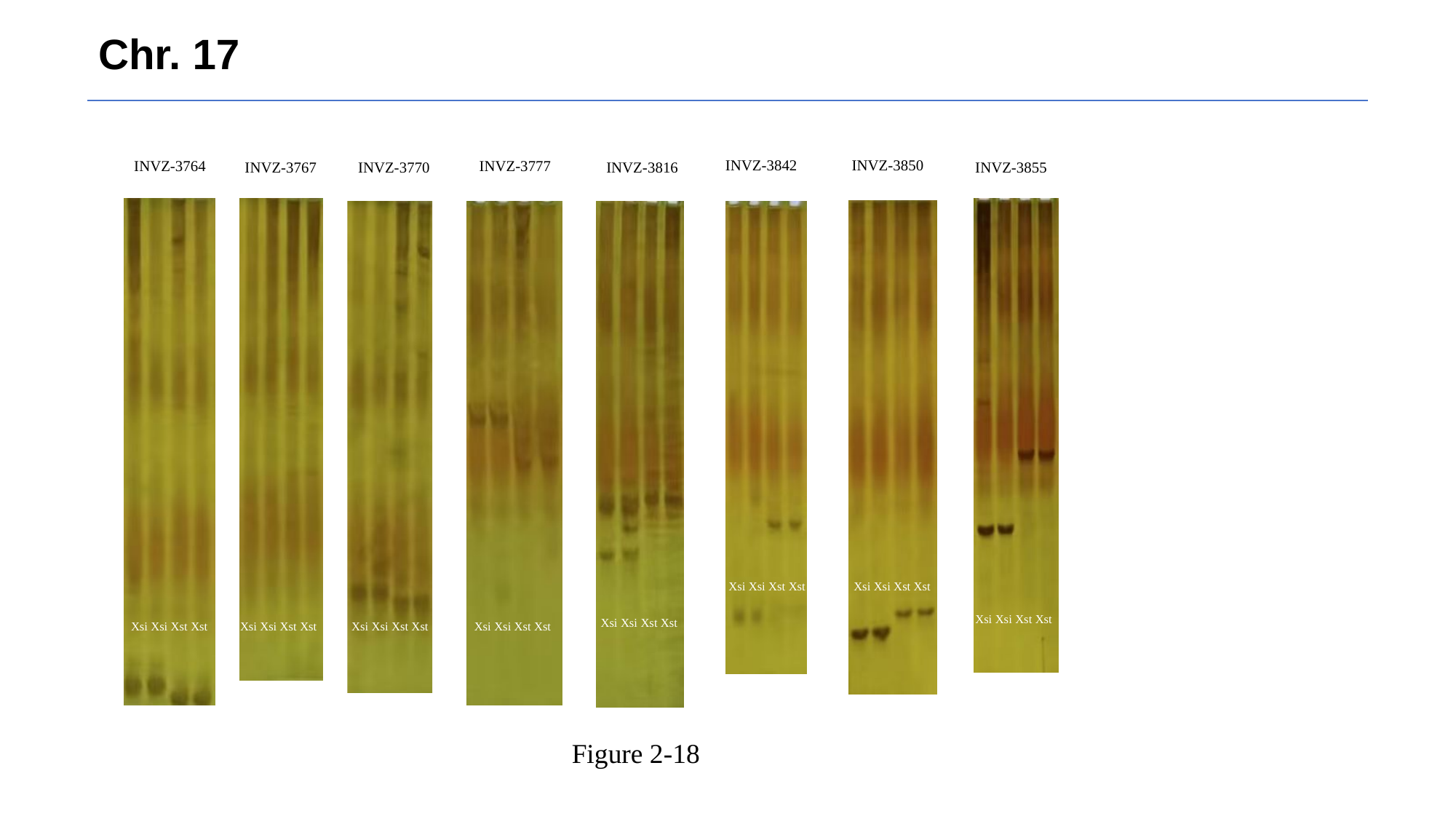

Chr. 17
INVZ-3850
INVZ-3842
INVZ-3764
INVZ-3777
INVZ-3855
INVZ-3767
INVZ-3770
INVZ-3816
Xsi Xsi Xst Xst
Xsi Xsi Xst Xst
Xsi Xsi Xst Xst
Xsi Xsi Xst Xst
Xsi Xsi Xst Xst
Xsi Xsi Xst Xst
Xsi Xsi Xst Xst
Xsi Xsi Xst Xst
Figure 2-18
