# Supplementary figures and images for: Genome-Wide Development and Characterization of 169 gSSR Markers in the Invasive Plant Xanthium strumarium L
Source: Plants (Basel). 2025 Nov 18;14(22):3522. doi: 10.3390/plants14223522 (PMC12656314; doi:10.3390/plants14223522)

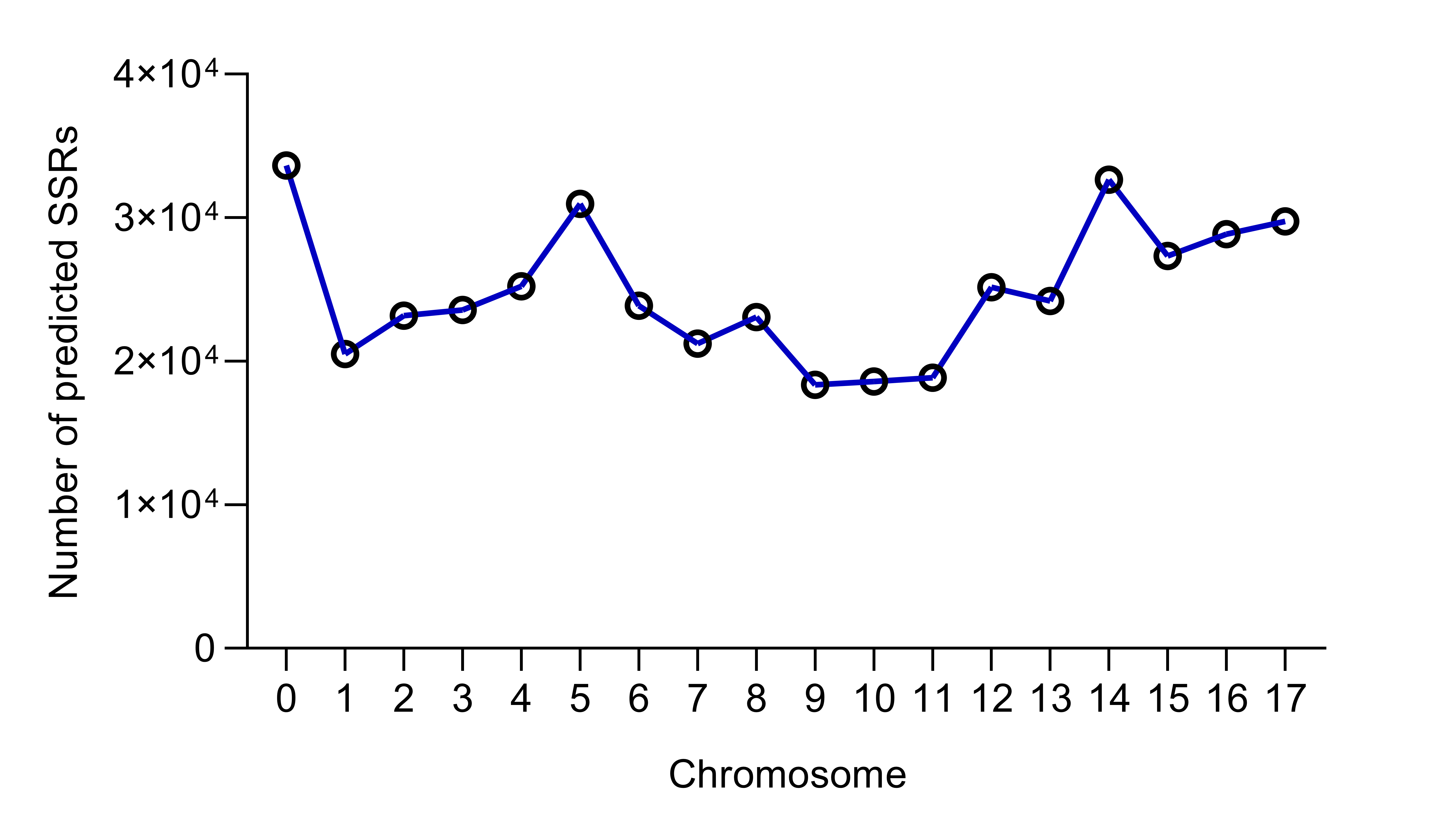

Supplement: Supplementary file 1 [file plants-14-03522-s001.zip › Fig. S1.tif]
